# *H. pylori*‐induced NF‐κB‐PIEZO1‐YAP1‐CTGF axis drives gastric cancer progression and cancer‐associated fibroblast‐mediated tumour microenvironment remodelling

**DOI:** 10.1002/ctm2.1481

**Published:** 2023-11-20

**Authors:** Bonan Chen, Xiaoli Liu, Peiyao Yu, Fuda Xie, Johnny S. H. Kwan, Wai Nok Chan, Canbin Fang, Jinglin Zhang, Alvin H. K. Cheung, Chit Chow, Gloria W. M. Leung, Kam Tong Leung, Shihua Shi, Bin Zhang, Shouyu Wang, Dazhi Xu, Kaili Fu, Chi Chun Wong, William K. K. Wu, Michael W. Y. Chan, Patrick M. K. Tang, Chi Man Tsang, Kwok Wai Lo, Gary M. K. Tse, Jun Yu, Ka Fai To, Wei Kang

**Affiliations:** ^1^ Department of Anatomical and Cellular Pathology State Key Laboratory of Translational Oncology Sir Y.K. Pao Cancer Center Prince of Wales Hospital The Chinese University of Hong Kong Hong Kong China; ^2^ Institute of Digestive Disease State Key Laboratory of Digestive Disease Li Ka Shing Institute of Health Science The Chinese University of Hong Kong Hong Kong China; ^3^ CUHK‐Shenzhen Research Institute Shenzhen China; ^4^ Department of Pathology School of Basic Medical Sciences Southern Medical University Guangzhou China; ^5^ Department of Pediatrics The Chinese University of Hong Kong Hong Kong China; ^6^ Hospital of Chengdu University of Traditional Chinese Medicine Chengdu China; ^7^ Friedrich Miescher Institute for Biomedical Research Basel Switzerland; ^8^ Department of Gastroenterology Nanjing Drum Tower Hospital The Affiliated Hospital of Nanjing University Medical School Nanjing China; ^9^ Department of Hepatobiliary Surgery The Affiliated Drum Tower Hospital of Nanjing University Medical School Nanjing China; ^10^ Department of Gastric Surgery Department of Oncology Fudan University Shanghai Cancer Center Shanghai Medical College Fudan University Shanghai China; ^11^ Department of Medicine and Therapeutics The Chinese University of Hong Kong Hong Kong China; ^12^ Department of Anaesthesia and Intensive Care The Chinese University of Hong Kong Hong Kong China; ^13^ Department of Life Science National Chung Cheng University Chiayi Taiwan

**Keywords:** cancer‐associated fibroblast, CTGF, gastric cancer, *H*. *pylori*, NF‐κB, PIEZO1, YAP1

## Abstract

**Background:**

Gastric cancer (GC) is one of the most common tumours in East Asia countries and is associated with *Helicobacter pylori* infection. *H. pylori* utilizes virulence factors, CagA and VacA, to up‐regulate pro‐inflammatory cytokines and activate NF‐κB signaling. Meanwhile, the PIEZO1 upregulation and cancer‐associated fibroblast (CAF) enrichment were found in GC progression. However, the mechanisms of PIEZO1 upregulation and its involvement in GC progression have not been fully elucidated.

**Methods:**

The CAF enrichment and clinical significance were investigated in animal models and primary samples. The expression of NF‐κB and PIEZO1 in GC was confirmed by immunohistochemistry staining, and expression correlation was analysed in multiple GC datasets. GSEA and Western blot analysis revealed the YAP1‐CTGF axis regulation by PIEZO1. The stimulatory effects of CTGF on CAFs were validated by the co‐culture system and animal studies. Patient‐derived organoid and peritoneal dissemination models were employed to confirm the role of the PIEZO1‐YAP1‐CTGF cascade in GC.

**Results:**

Both CAF signature and PIEZO1 were positively correlated with *H. pylori* infection. PIEZO1, a mechanosensor, was confirmed as a direct downstream of NF‐κB to promote the transformation from intestinal metaplasia to GC. Mechanistic studies revealed that PIEZO1 transduced the oncogenic signal from NF‐κB into YAP1 signaling, a well‐documented oncogenic pathway in GC progression. PIEZO1 expression was positively correlated with the YAP1 signature (CTGF, CYR61, and c‐Myc, etc.) in primary samples. The secreted CTGF by cancer cells stimulated the CAF infiltration to form a stiffened collagen‐enrichment microenvironment, thus activating PIEZO1 to form a positive feedback loop. Both PIEZO1 depletion by shRNA and CTGF inhibition by Procyanidin C1 enhanced the efficacy of 5‐FU in suppressing the GC cell peritoneal metastasis.

**Conclusion:**

This study elucidates a novel driving PIEZO1‐YAP1‐CTGF force, which opens a novel therapeutic avenue to block the transformation from precancerous lesions to GC. *H. pylori*‐NF‐κB activates the PIEZO1‐YAP1‐CTGF axis to remodel the GC microenvironment by promoting CAF infiltration. Targeting PIEZO1‐YAP1‐CTGF plus chemotherapy might serve as a potential therapeutic option to block GC progression and peritoneal metastasis.

## Graphical abstract

**Description**:

1. *H. pylori* induced NFκB directly regulates PIEZO1 transcription in gastric cancer (GC).

2. *H. pylori* prompts α‐SMA^+^ CAF accumulation in GC, driven by PIEZO1/YAP1/CTGF axis activation.

3. Enhanced microenvironment stiffness from augmented α‐SMA^+^ CAFs perpetuates PIEZO1 activation in a deleterious loop in GC.

4. Targeting CTGF with Procyanidin C1 emerges as a potential therapeutic strategy for *H. pylori*‐associated GC.

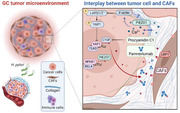

## 1 INTRODUCTION

According to the Global Cancer Statistics 2020 (GLOBOCAN), gastric cancer (GC) ranks as the fifth most commonly diagnosed malignancy and the fourth leading cause of cancer‐related deaths globally.^1^ *Helicobacter pylori*, a well‐established carcinogenic pathogen, accounts for 65%−80% of primary GCs, predominantly causing inflammation within the gastric epithelium.^2^ It employs virulence factors, including CagA, VacA and peptidoglycan, to elevate the levels of pro‐inflammatory cytokines such as Interleukin‐1 (IL‐1), IL‐6, IL‐8, and tumor necrosis factor‐α (TNF‐α), thereby activating the NF‐κB signalling cascade within gastric epithelial cells.^3^ Consequently, the chronic inflammation mediated by *H. pylori*‐induced NF‐κB becomes pivotal for both the initiation and progression of GC. The evolution of GC is sequential, transitioning from normal gastric mucosa to non‐atrophic gastritis, then to intestinal metaplasia (IM), dysplasia, and eventually culminating in GC.^4^ Among these stages, IM, especially its incomplete and extensive forms, is viewed as the most critical juncture leading to GC.^5^, ^6^ Correlation between NF‐κB activation and IM has been observed in *H. pylori*‐infected patients, and its eradication has been shown to stabilize the risk, slowing the progression towards GC.^7^

In a preceding study, we were the first to elucidate the clinical relevance of PIEZO1 in GC.^8^ Specifically, PIEZO1 is found to be prominently expressed in numerous GC cell lines and primary samples. Elevated levels of PIEZO1 correlate with unfavorable disease‐specific outcomes. Diminishing PIEZO1 levels induces an anti‐tumour response and amplifies sensitivity to therapeutics like Cisplatin and 5‐fluorouracil (5‐FU). PIEZO1 also modulates Rho GTPase activity, which facilitates cell invasion and migration. Significantly, the interaction between PIEZO1 and TFF1 proteins mitigates nestin loss‐induced apoptosis and enhances motility in GC cells, consequently augmenting the invasive and migratory capacities.^9^ Moreover, PIEZO1 can orchestrate cellular adaptation to hypoxic conditions by elevating the expression of hypoxia‐inducible factor 1‐alpha in GC cells,^10^ thus fostering cell migration and Calpain1/2 expression. Research has revealed that the activation of PIEZO1 elevates mitochondrial membrane potential (Ψm) and intracellular Ca^2+^ levels in GC cells, with Ψm serving a pivotal role in cancer metastasis and angiogenesis. In vitro analyses also demonstrated that interference with PIEZO1 significantly downregulates the expression of P53/P21 axis. Consequently, PIEZO1 emerges as a potential key regulator in modulating the proliferation of GC cells.

Extracellular matrix (ECM) remodeling and sclerosis are characteristic of solid tumours, and tissue hardness has been used to detect various human cancer types. Of note, mechanically stiff microenvironments play a pivotal role in tumourigenesis and tumour progression. ECM that surrounds cells can undergo various biophysical changes during the development and progression of cancer, resulting in increased rigidity or stiffness. This stiffness can modulate cellular behavior and fate, influencing processes such as cell proliferation, migration, and differentiation. Mechanotransduction pathways, whereby cells sense and respond to mechanical cues, are activated in response to this altered mechanical environment. Importantly, cells in a stiff microenvironment often exhibit enhanced invasive and migratory capabilities, which are hallmark characteristics of aggressive tumours. Additionally, a stiff matrix can lead to changes in cellular morphology and promote a phenotypic switch to a more mesenchymal, migratory state, further driving metastasis. Emerging evidence also suggests that this stiff microenvironment can influence the tumour immune response and drug resistance. Conventionally recognised as a mechanosensor at the context of tumour microenvironment,^11^ PIEZO1's interaction with α‐smooth muscle actin (SMA) cancer‐associated fibroblasts (CAFs)‐hybrid cells exhibiting characteristics of both fibroblasts and smooth muscle cells‐raises intrigue. These myofibroblast CAFs produce ECM and induce mechanical tension within tumours via cellular contraction.^12^, ^13^ However, the interplay between α‐SMA^+^ CAFs and PIEZO1 within cancer cells, including its activation mechanism, subsequent signaling pathways, and role in GC microenvironmental alteration, remains to be uncovered.

In our current research, we aim to define the direct modulation of PIEZO1 via *H. pylori*‐induced NF‐κB activation. Concurrently, we will uncover the YAP1‐connective tissue growth factor (CTGF) axis as the paramount downstream conduit of PIEZO1. Ultimately, we will verify that the CTGF secretion triggers the infiltration of α‐SMA^+^ CAFs, fostering a mechanically stiff microenvironment conducive for PIEZO1 activation.

## 2 MATERIALS AND METHODS

### 2.1 GC cell lines, CAFs, organoids, primary samples and clinical cohorts

In this study, we employed five GC cell lines, MKN28, NCI‐N87, GSE1, MKN1 and AGS, obtained from the American Type Culture Collection (ATCC, Manassas, VA). These cell lines were cultured in RPMI‐1640 medium (Life Technologies, NY, USA), supplemented with 10% Fetal Bovine Serum (FBS, Life Technologies, NY, USA) and 1% penicillin‐streptomycin (PS, catalog number #15240062, Life Technologies, NY, USA). NCI‐N87 cells were specifically transduced with a lentivirus carrying a firefly luciferase expression construct (Addgene, Cambridge, MA, USA), and selected using puromycin (1 μg/mL; Life Technologies, California, USA) for 2 days as per protocol.^14^ Concurrently, CAFs were isolated from clinical GC samples and maintained in advanced DMEM/F12 medium (Life Technologies, NY, USA) enriched with 10% FBS. Organoid cultures were derived from GC biopsies, with cells isolated and cultured in accordance with established protocol.^15^ Tumor tissues, ranging in size from .5 to 1 cm^3^, were rinsed, minced and incubated in a collagenase‐accutase digestion solution at 37°C for 1 h. The digestion process was terminated with cold culture medium, and the cellular suspension was filtered through a 70 μm strainer and centrifuged at 400 g for 5 min. The collected cell pellet was subsequently integrated with Matrigel (BD Biosciences, California, USA) to establish a 3D droplet culture model. We also analyzed two patient cohorts: the Hong Kong cohort with 278 samples and the Beijing cohort with 162 cases, both collected between 2002 and 2014. Ethical approval for the use of human samples was granted by the Joint Chinese University of Hong Kong‐New Territories East Cluster Clinical Research Ethics Committee. Additional data were sourced from The Cancer Genome Atlas (TCGA) GC cohort, featuring 375 GC cases and accessible via its official website, as well as from the ACRG GC cohort, which includes 300 GC cases extracted from dataset GSE62254 (refer to Table S1 for data sources).

### 2.2 Immunohistochemistry (IHC), Masson's trichrome staining, and Van Gieson's staining

Immunohistochemistry (IHC) was performed on tissue microarray samples using the Ventana NexES Automated Stainer (Ventana Corporation). Following deparaffinization, 5 μm tissue sections underwent antigen retrieval using an EDTA antigen retrieval solution (catalog number #P0086, Beyotime, Shanghai, China). The sections were then incubated with primary antibodies overnight at 4°C, followed by exposure to horseradish peroxidase (HRP)‐conjugated secondary antibodies. Details of the primary and secondary antibodies are provided in Table S2. Protein expression in the tissue sections was visualized using the Liquid DAB^+^ Substrate Chromogen System kit (catalog number #K3468, Dako North America, California, USA) and counterstained with hematoxylin (catalog number #G1004, Servicebio, Wuhan, China). The immunoreactive score for protein expression was quantified based on the percentage of positively stained tumour cells and staining intensity, and analyses were conducted using ImageJ software. In addition to IHC, Masson's trichrome and Van Gieson's stains were employed to visualize collagen and connective tissues, respectively. For Masson's trichrome staining, samples were prepared using the Masson Tricolor Staining Solution kit (catalog number #G1343, Solarbio, Beijing, China). The Elastica van Gieson staining kit (catalog number #115974, Sigma‐Aldrich, MO, USA) was utilized for the Van Gieson staining procedure.

### 2.3 Protein extraction and Western blot analysis

Cell lysates were prepared by incubating cells on ice for 30 min in RIPA lysis buffer. Subsequently, the total protein concentration was quantified using the BCA Protein Assay Kit (Cat. 23225, Thermo Scientific, Waltham, MA, USA). In accordance with previously published protocol,^16^ protein samples were separated using sodium dodecyl sulfate‐polyacrylamide gel electrophoresis (SDS‐PAGE) and then transferred onto polyvinylidene fluoride (PVDF) membranes, which were subsequently blocked for 2 h in a Tris‐buffered saline with Tween (TBST) buffer supplemented with 5% skim milk. Following this, the membranes were incubated overnight at 4°C with primary antibodies. The membranes were then washed twice with TBST and incubated for an additional hour with appropriate HRP‐conjugated secondary antibodies. Protein bands were visualized using enhanced chemiluminescence. Specific details of the primary and secondary antibodies employed for these assays have been provided in Table S3.

### 2.4 Immunofluorescence analysis

Cell slides were initially fixed with 4% paraformaldehyde (PFA) for 15 min at room temperature. They were then blocked for 2 h in a Phosphate buffer saline (PBS) buffer containing .1% Triton X‐100 (Catalog number X100, Sigma‐Aldrich, MO, USA) and 1% bovine serum albumin (#81‐003‐3, Millipore, Boston, MA, USA). Subsequently, the slides were incubated overnight at 4°C with primary antibodies, followed by a 1‐h incubation at room temperature with appropriate secondary antibodies. Details of the primary and secondary antibodies are provided in Table S4. For nuclear staining, the sections were treated with 1 μg/mL DAPI (#MBD0015, Sigma‐Aldrich, MO, USA) for 15 min at room temperature. Imaging was performed using a Zeiss laser scanning microscopy (LSM) 880 confocal microscope.

### 2.5 Multiplex fluorescent IHC

Tissue microarray sections were first deparaffinized and underwent antigen retrieval using an EDTA antigen retrieval solution. Following a brief 5‐min blocking step, the sections were incubated overnight at 4°C with primary antibodies, and subsequently with HRP‐conjugated secondary antibodies. Chromogenic fluorescent staining was then applied. If additional protein staining was required, antigen retrieval was repeated, and the aforementioned steps were carried out again for the second protein. Sections were finally treated with 1 μg/mL DAPI (#MBD0015, Sigma‐Aldrich, MO, USA) for 15 min at room temperature for nuclear staining. The specific primary and secondary antibodies utilized in these experiments have been listed in Table S5. Imaging was conducted using the Mantra system, and fluorescence quantification was performed with inForm software.

### 2.6 In vitro functional assays

For cell transfection, siNFKB1 (SI02654932 and SI02662618), siRELA (SI00301672 and SI04437062) and siPIEZO1 (SI04759167 and SI00383656) were purchased at Qiagen (Valencia, CA). Transfections were carried out using Lipofectamine 3000 Transfection Reagent (Thermo Fisher Scientific). Subsequent evaluation of cell proliferation was conducted using a variety of assays, including the Cell Counting Kit‐8 (CCK8, Tojindo, Japan), monolayer colony formation assays and cell invasion assays (Catalog number 354480, Corning, NY). These assays were performed in accordance with methodologies described in our previous study, referenced as protocol.^17^

### 2.7 Expression correlation and signalling pathway analysis

In the TCGA dataset, we conducted analyses to uncover the expression correlation between NF‐κB, PIEZO1, YAP1 signatures, and the CAF signature. Within the Hong Kong GC cohort, correlations among relevant biomarkers were assessed at the protein level using immunohistochemistry (IHC). Cases exhibiting high and low PIEZO1 expression levels were further examined through Gene Set Enrichment Analysis (GSEA) to elucidate the pathophysiological roles and potential signaling pathways involved. Additionally, scRNA‐seq analyses were carried out on primary GC samples to identify cell populations enriched for PIEZO1 and YAP1 signatures.^18^

### 2.8 Single‐cell RNA‐seq (scRNA‐seq) analysis

The scRNA‐seq dataset source is listed in Table S1. Most of the scRNA analysis was done using the R package ‘Seurat’ (version 4.0.2). After data cleaning and normalization, single‐cell gene expression was visualized using the functions ‘FeaturePlot’, ‘VlnPlot’ and ‘DotPlot’. Additionally, single‐cell pseudotime analysis was performed using Monocle2,^19^ which could be found under the R package ‘monocle’ (version 2.18.0). After creating the monocle subject using the function ‘newCellDataSet’, genes were filtered using recommended parameters for downstream analysis. The function ‘reduceDimension’, using the parameters reduction_method  =  “DDRTree” and max_components  =  2, was employed to reduce dimensions. The cells were ordered and visualized with the functions ‘orderCells’ and ‘plot_cell_trajectory’.

### 2.9 RNA extraction and RNA‐seq analysis

Cultured cells were harvested, and total RNA was extracted by RNeasy kit (Qiagen, Valencia, CA). RNA quality was assessed by Tapestation (Agilent, USA). The library was prepared by Illumina Truseq RNA Kit (Illumina, San Diego, CA, USA). RNA sequencing was conducted after PIEZO1 knockdown or activation by the NovaSeq 6000 platform (Illumina, San Diego, CA, USA) with single end read 100 bp. Reads were quality‐checked with FastQC (v0.12.0), and Cutadapt (v4.2) was used for sequence trimming. The duplicate reads were not filtered before the sequence read alignment. The raw sequencing reads were aligned to the Homo sapiens genome assembly GRCh37 (hg19) from NCBI database using STAR (v2.5.3a). Gene expressions were quantified by Cufflinks (v2.2.1) and indicated by Fragments per kilobase of transcript per million mapped reads (FPKM).^20^ Further, the differentially expressed genes (DEGs) were identified using the R package “limma” (v3.46.0, |log2 (fold change)| > 1), and then the GO enrichment was performed by R package ‘clusterProfiler’ (v4.0.5). Meanwhile, the heatmap was drawn using the R package ‘pheatmap’ (v1.0.12) to show the variations in the expression of YAP1‐related genes. The raw data were submitted to the Gene Expression Omnibus under the accession numbers GSE228690 and GSE228691 (Table S1). Besides, the cDNA synthesis was performed using a High‐Capacity cDNA Reverse Transcription Kit (Applied Biosystems, Carlsbad, CA, USA). Quantitative real‐time polymerase chain reaction (RT‐qPCR) was applied to detect mRNA expression levels of candidate genes screened in the RNA‐seq analysis. All primer sequences were listed in Table S6.

### 2.10 ChIP‐qPCR and dual‐luciferase assay

Crosslinked chromatin samples were sheared by a Covaris sonication system (S220) to desired fragments (100‐500 bp). The sonicated products were incubated with Magnetic Dynabeads Protein G (10003D, Thermo Fisher, Waltham, MA) and linked with anti‐RELA (#3034, Cell Signaling) or Normal anti‐IgG antibody (#2729, Cell signaling). The antibodies for ChIP‐qPCR were listed in Table S7. According to our previously published protocol,^21^ for qPCR, equivalent quantities of IP (by RELA antibody or IgG control) were set for routine PCR assays using specific primers targeting the region within 100 bp from the putative binding site (primers are listed in Table S8). For the dual‐luciferase assay, cells were transiently co‐transfected with either reporter plasmid or a mutant counterpart to confirm the promoter binding affinities for the transcription factors. Sequences of the putative binding regions and mutations were listed in Table S9.

### 2.11 Isolation and identification of CAFs

Clinical GC tissue samples were initially rinsed three times with a PBS solution containing 1% PS. Subsequently, the tissue blocks were dissected into 1 mm^3^ pieces using sterile scissors. These pieces were then treated with type I collagenase solution (Sigma) and incubated in a 5% CO₂ environment at 37°C for 20 min, or until the tissue blocks were fully digested and dispersed into a single‐cell suspension. The resulting cell suspension was centrifuged and washed with PBS for further processing. Cells were subsequently cultured in a specialized medium optimized for CAF growth. After a designated period of culture, isolation of the GC CAF cell line was confirmed through the detection of CAF‐specific markers, including ATCA2, FAP and VIM, via qRT‐PCR.

### 2.12 Yoda1 stimulation and intracellular Ca^2+^ imaging

NCI‐N87 cells were transfected with pGP‐CMV‐GCaMP6f (Plasmid #40755, Addgene) expressing calcium sensor to represent intracellular Ca^2+^ responses. PIEZO1 agonist Yoda1 (40 μM) was added at Second 150, and Ca^2+^ (2 mM) was restored 60 s later. To block the activation of PIEZO1, GsMTx4 (5 μM, HY‐P1410, MedChemExpress, MCE) was added in Ca^2+^‐free buffer prior to fluorescence recording. The Ca^2+^ response was expressed as a ration of fluorescence intensities relative to the basal intensities prior to extracellular Ca^2+^ influx.

### 2.13 Molecular docking

The CTGF 3D structure file was downloaded from AlphaFold (https://alphafold.ebi.ac.uk/).^22^ The compound library was composed of 4511 anti‐cancer small molecules, while their structure information was downloaded from PubChem^23^ (https://pubchem.ncbi.nlm.nih.gov/). Additionally, the active CTGF sites were predicted by PrankWeb (https://prankweb.cz/) and DeepSite (https://www.playmolecule.com/deepsite/).^24^ Molecular docking was performed by Autodock 4.2.6 to predict the binding condition between the predicted sites among the Procyanidin C1 and CTGF.^25^ The 3D structure of CTGF and binding site analysis of Procyanidin C1 were visualised by PyMOL 2.3. The docking procedures used the Lamarckian genetic algorithm, and the parameters were as follows: energy grid box, 40 × 40 × 40 Å; energy grid spacing, .375 Å; the number of individuals in a population, 150; the maximum number of energy evaluations, 2.5 × 10^6^; the maximum number of generations, 2.7 × 10^4^; rate of gene mutation, .02. The docking results were evaluated by the calculated binding energy (BE). Compounds with BE lower than −9.549 kcal/mol (representing the inhibition constant as .1 μM, Ki < 10^−7^) were shown in Table S10.

### 2.14 Animal studies

The BALB/c nude and NOD scid gamma (NSG) mice were employed for the animal studies. For the PIEZO1 study (*n* = 5/group). The control group was peritoneally inoculated with NCI‐N87 cells (10^5^ cells/mouse), and the second group received NCI‐N87 cells with PIEZO1 overexpression. The tumour nodules were taken out after 28 days for examination and counting. For evaluating the synergistic effect of PIEZO1 depletion and 5‐FU, four groups of mice (*n* = 5/group, NCI‐N87 peritoneal inoculation mice) were administrated with vehicle (.1% DMSO), shPIEZO1, 5‐FU (8 mg/kg/2day), shPIEZO1 + 5‐FU, respectively for 4 weeks. 5‐FU (MedChemExpress, NJ, USA) was administered via intraperitoneal (i.p.) injection. The mice were sacrificed after 28‐day administration, and the peritoneum nodules were taken out for counting. All cell lines with overexpression or knockdown of genes were constructed in vitro and subsequently utilized for animal experiments.

To investigate the efficacy of CTGF inhibitor combined with 5‐FU in GC peritoneal metastasis, we mixed CAFs (10^5^ cells/mouse) and NCI‐N87‐luciferase (10^5^ cells/mouse) and then injected them into the peritoneal cavity of NSG mice (*n* = 5/group). The combinational administration of Pamrevlumab, Procyanidin C1, and 5‐FU in controlling GC peritoneal metastasis. Total six groups of mice were administrated with vehicle (.1% DMSO), 5‐FU (8 mg/kg/2days), Procyanidin C1 (20 mg/kg/2days), 5‐FU + Procyanidin C1, Pamrevlumab (10 mg/kg/2day), Pamrevlumab + 5‐FU respectively for 60 days. GC peritoneal metastases were monitored using the IVIS 200 in vivo imaging system (Xenogen, Alameda, CA, USA) on day 14 after treatment. Before imaging, mice were injected with D‐fluorescein (150 mg/kg; Promega, Madison, WI, USA) and anesthetized with 2.5% isoflurane (Zoetis, Parsippany, NJ, USA). The luminescence signal was analyzed by Living Image software (Xenogen) as photon emission/sec/cm^2^. The time to death of NSG mice was also recorded during the whole procedure. All mice experiments were authorized by Animal Ethics Experimentation Committee (AEEC) from CUHK.

### 2.15 Statistical analysis

Student's *t*‐test was used to compare the expression levels and the functional variations between assay groups and control. Spearman's correlation evaluated correlation analyses. Statistical analyses were performed by GraphPad Prism 8.0 (GraphPad, San Diego, CA) and SPSS software (Version 22.0; SPSS Inc). Data were expressed as mean ± standard error of the mean (SEM) of triplicate independent experiments. Two‐tailed *p*‐value of less than .05 was defined as statistically significant, and *p*‐value of less than .001 was considered highly significant.

## 3 RESULTS

### 3.1 *H. pylori*‐positive GC cases demonstrate an abundance of α‐SMA^+^ CAFs infiltration

**FIGURE 1:**
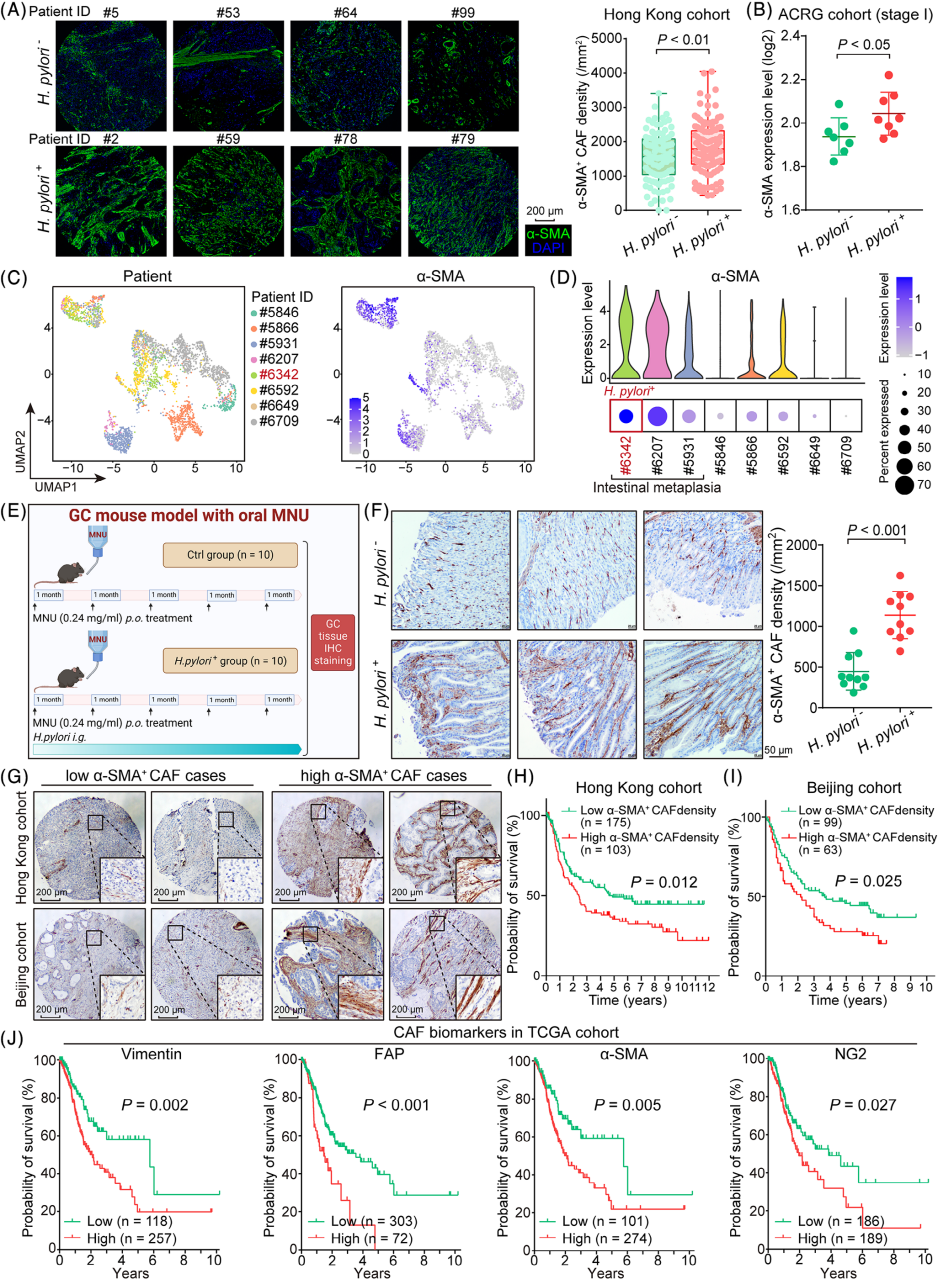
*H. pylori* infection increases the abundance of α‐SMA^+^ cancer‐associated fibroblasts (CAFs) in gastric intestinal metaplasia. (A) The representative immunohistochemistry images on the left depict distinct variations in α‐SMA expression between *H. pylori* ^+^ and *H. pylori* ^−^ gastric cancer (GC) patients. Specifically, α‐SMA^+^ CAF density was assessed in 103 *H. pylori* ^−^ and 121 *H. pylori* ^+^ GC patients (scale bar, 200 μm). (B) Expression levels of α‐SMA mRNA were examined in stage I GC patients who did not receive concurrent adjuvant chemotherapy, as per the ACRG dataset, which included 7 *H. pylori* ^−^ and 8 *H. pylori* ^+^ GC patients. (C) A UMAP plot illustrates the distribution of α‐SMA mRNA expression in GC patients based on a scRNA‐seq dataset. (D) Violin and bubble plots reveal elevated expression levels of α‐SMA in scRNA‐seq samples of GC intestinal metaplasia, particularly in the *H. pylori* ^+^ sample (#6342). (E) A schematic diagram outlines a GC mouse model, which demonstrates the formation of intestinal metaplasia in situ in C57BL/6 mice after oral administration of MNU. A second group of mice was treated with *H. pylori* (*n* = 10 for each group). (F) Representative immunohistochemical images display α‐SMA staining in both *H. pylori* ^−^ and *H. pylori* ^+^ GC intestinal metaplasia in mice (scale bar, 50 μm). Accompanying scatter plots quantify the α‐SMA^+^ CAF density in both sets of mice. (G) IHC images of low‐density and high‐density α‐SMA^+^ CAF cases in the Hong Kong and Beijing GC cohorts (scale bar, 200 μm). (H‐I) α‐SMA^+^ CAF density predicted poor survival in GC patients (Hong Kong cohort, *n* = 278, *p* = .012; Beijing cohort, *n* = 162, *p* = .025). (J) Upregulation of CAF biomarkers, including Vimentin, FAP, α‐SMA, and NG2 was associated with unfavorable clinical outcomes (TCGA cohort, *n* = 375).

We conducted α‐SMA immunohistochemistry on the tissue microarray and observed that *H. pylori* ^+^ GC cases exhibited a higher density of α‐SMA^+^ CAFs in comparison to *H. pylori* ^−^ GC cases (Figure 1A; Table S11). Moreover, among stage I GC patients who did not receive concurrent adjuvant chemotherapy according to the ACRG dataset, we noted that 8 *H. pylori^+^* GC patients displayed abundant CAF infiltration compared to 7 *H. pylori* ^−^ GC patients, as evidenced by α‐SMA expression (Figure 1B). This was further corroborated by single‐cell RNA sequencing (scRNA‐seq) analysis, where the *H. pylori* ^+^ GC case exhibited a notably elevated expression of α‐SMA (Figure 1C). Given that IM is a precursor lesion of GC and is linked to *H. pylori* infection, we extended our investigation to evaluate α‐SMA expression in the 8 GC scRNA‐seq samples. Notably, α‐SMA was prominently expressed in GC samples featuring IM, particularly in the *H. pylori^+^* sample (#6342), thereby suggesting an association between α‐SMA^+^ CAF infiltration and *H. pylori* infection (Figure 1D).

Subsequently, an animal model was employed to validate the influence of *H. pylori* infection on CAF infiltration. By administering MNU via gavage to C57BL/6 mice, with or without concurrent *H. pylori* exposure, we observed the development of IM accompanied by in situ GC within the stomach tissues of these mice (Figure 1E). Through α‐SMA immunohistochemical staining, it became evident that mice subjected to *H. pylori* ^+^ gavage displayed a substantial influx of α‐SMA^+^ CAFs within the stomach epithelial tissues (n = 10/group; Figure 1F). Furthermore, we undertook a quantification of low‐density and high‐density SMA^+^ CAFs in cohorts from Hong Kong and Beijing (Figure 1G). Notably, cases characterized by a high α‐SMA^+^ CAF density demonstrated an association with unfavorable prognoses (Hong Kong cohort: *n* = 278, *p* = .012, Figure 1H; Beijing cohort: *n* = 162, *p* = .025, Figure 1I). Additionally, there was a consistent correlation between the upregulation of classical CAF biomarkers, such as Vimentin, FAP, α‐SMA, and NG2, and adverse clinical outcomes (TCGA cohort, *n* = 375, Figure 1J).

Considering that GC can be classified into intestinal and diffuse types, we proceeded to compare the CAF signature between these two categories. Notably, the diffuse type of GC exhibited a poorer prognosis compared to the intestinal type across the Hong Kong, TCGA, and ACRG cohorts (Figure S1A‐C). This finding was further substantiated by a significant correlation between the diffuse type and the expression of CAF signature genes (*ACTA2, RGS5, MYH11, FAP, etc*.), metastasis, and RHOA/CDH1 mutation (Figure S1D). Furthermore, the expression of CAF biomarkers, including VIM, FAP, α‐SMA and NG2, was notably higher in the diffuse type compared to the intestinal type (Figure S1E), with elevated FAP expression being specifically associated with a poorer prognosis in diffuse type GC cases (Figure S1F). Collectively, these findings underline the role of *H. pylori* infection in fostering α‐SMA^+^ CAF infiltration, thereby serving as a prognostic indicator of adverse outcomes in GC.

### 3.2 *H. pylori*‐induced NF‐κB activation upregulates PIEZO1 expression in GC progression

**FIGURE 2:**
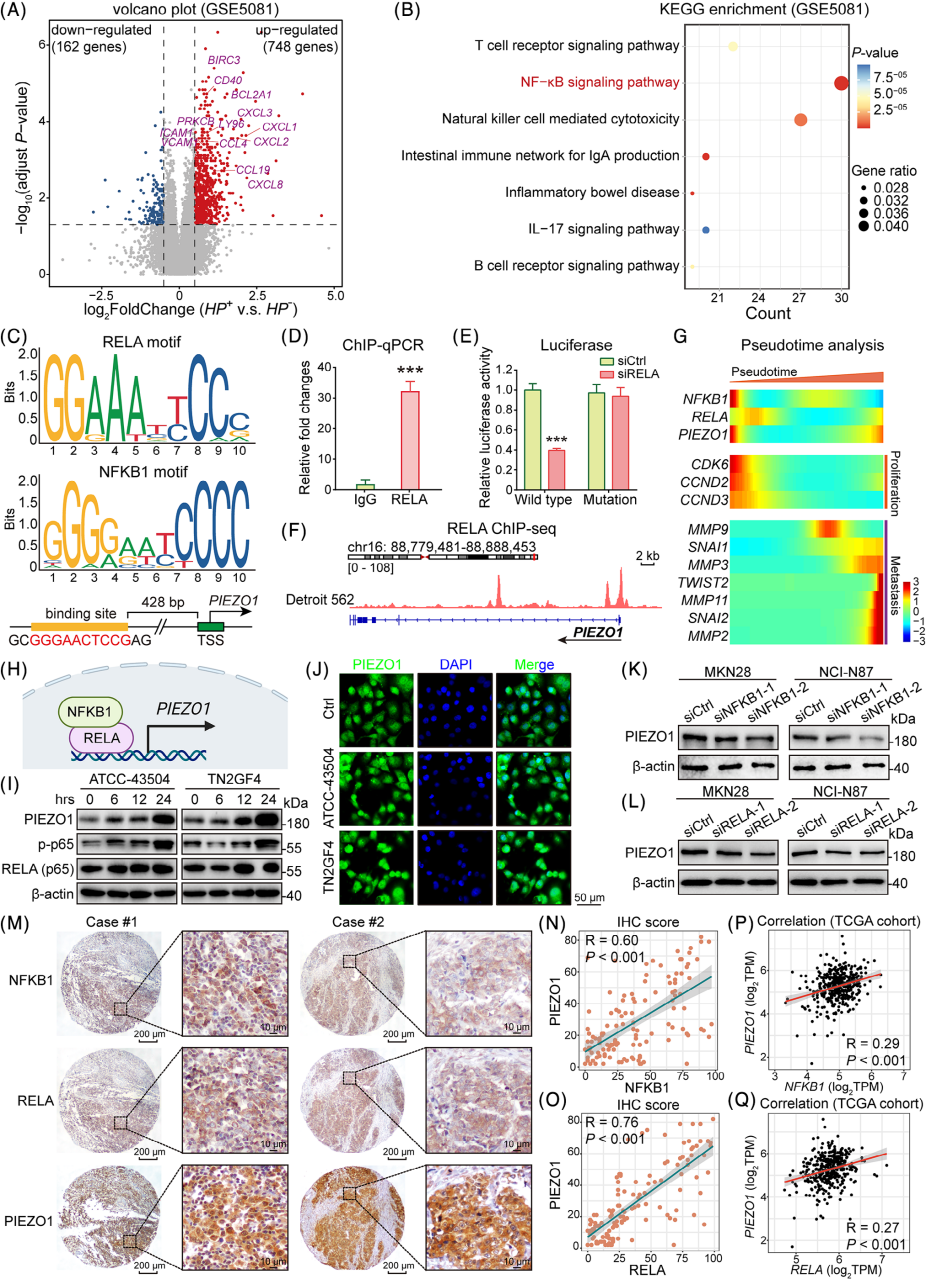
*H. pylori*‐induced activation of NFKB1/RELA directly regulates the transcriptional expression of PIEZO1 in gastric cancer (GC). (A) A Volcano plot illustrates elevated expression levels of inflammation‐associated genes in *H. pylori*‐activated GC. (B) KEGG enrichment analysis of DEGs highlights inflammation‐related pathways, notably the NF‐κB signaling pathway. (C) JASPAR‐predicted NFKB1/RELA binding motif is identified in the PIEZO1 promoter, suggesting a regulatory relationship. (D) ChIP‐qPCR using RELA antibody pulldown. (E) Duo‐luciferase assays provide evidence that NF‐κB directly interacts with the PIEZO1 promoter to activate its expression. (F) RELA ChIP‐sequencing in Detroit 562 cells reveals a peak at the PIEZO1 promoter, further corroborating the binding of RELA to this region. (G) Pseudotime analysis indicates that NFKB1/RELA and PIEZO1 are co‐upregulated along with proliferation‐associated genes in the early stages of GC, followed by the upregulation of metastasis‐related genes. (H) A schematic diagram encapsulates the finding that NFKB1/RELA serves as the transcription factor for PIEZO1. (I) Exposure to *H. pylori* augments the phosphorylation level of p65, along with the expression of PIEZO1. (J) Immunofluorescence staining showed that *H. pylori* treatment increased the mean fluorescence of PIEZO1 (green) intensity (DAPI, blue; scale bar, 50 μm). (K and L) Knocking down NFKB1 and RELA decreased PIEZO1 expression in MKN28 and NCI‐N87. (M and O) The protein levels of NFKB1/RELA and PIEZO1 demonstrated a positive correlation in primary samples (scale bar, 200 μm for low magnification and 10 μm for high magnification). (P‐Q) PIEZO1 expression positively correlated with NFKB1 (P) and RELA (Q) from TCGA cohort, respectively.

To gain a deeper comprehension of the signaling pathways influenced by *H. pylori* infection in relation to GC, we delved into the GSE5081 dataset containing gastric mucosal and erosion data, segregating it into *H. pylori*‐positive and ‐negative groups. The positive group exhibited pronounced elevation in the expression of multiple cytokines and receptors linked to NF‐κB activation (Figure 2A). Notably, when compared to the *H. pylori*‐negative group, the up‐regulated genes were primarily enriched within the NF‐κB pathway and the natural killer cell‐mediated cytotoxicity pathway (Figure 2B). This observation was corroborated through IHC staining of NFKB1 and RELA protein expression in the original samples, revealing heightened expression in both IM and tumour samples in contrast to adjacent normal epithelial cells (Figure S2A‐B). Quantitative analysis further affirmed the activation of NFKB1 and RELA in IM and cancer cells, signifying their up‐regulation as a pivotal early event in the process of GC progression (Figure S2C). Analogously, an exploration of mRNA expression of NFKB1 and RELA, two canonical constituents of the NF‐κB pathway, across normal stomach epithelium (*n* = 20), IM (*n* = 20) and GC (*n* = 30) demonstrated heightened expression in IM and GC, particularly pronounced in IM samples (Figure S2D).

Subsequently, we selected two GC cell lines, MKN28 and NCI‐N87, characterized by elevated NFKB1 and RELA expression compared to other GC cell lines (Figure S4), for functional assessments. Evidently, knockdown of NFKB1 and RELA instigated an anti‐tumour effect by suppressing cell proliferation as evidenced by monolayer colony formation assay (Figure S5A,B) and by inhibiting cell invasion (Figure S5C,D). These effects were supplemented by Western blot analysis, unveiling alterations in cell cycle and apoptosis‐related biomarkers. G1 phase cell cycle arrest and advanced apoptosis stages were substantiated by increased levels of p21, p27 and cleaved‐PARP, accompanied by a decrease in CDK6 and pRb expression (Figure S5E‐F). Employing NFKB1/RELA binding motifs predicted by the Eukaryotic Promoter Database (https://epd.epfl.ch//index.php) and JASPAR 2022 database (https://jaspar.genereg.net/), a putative binding site was identified in the PIEZO1 promoter region at −428 bp, as illustrated in Figure 2C. This binding affinity was subsequently affirmed through ChIP‐qPCR utilizing RELA antibody pulldown (Figure 2D). Further corroborating this, Duo‐Luciferase assays elucidated that NF‐κB directly binds to the PIEZO1 promoter, thereby transcriptionally activating its expression; notably, mutation of this binding site abrogated this activation effect (Figure 2E). Through peak calling analysis on the ChIP‐seq dataset GSM2419824 derived from RELA antibody pull‐down, we identified a RELA binding region proximal to the transcription start site of PIEZO1. Significantly, in the early stages of tumour development, both NFKB1/RELA and PIEZO1 displayed heightened expression levels, paralleled by similar kinetic trends of proliferation‐associated genes such as CDK6, CCND1 and CCND3, as unveiled by functional pseudotime analysis of the scRNA‐seq dataset (Figure 2G). This strongly implies that NFKB1/RELA can trigger PIEZO1 transcription, thereby intricately linking it to GC progression (Figure 2H).

Lastly, to confirm the impact of *H. pylori* infection on the NFKB1/RELA/PIEZO1 pathway, we exposed GC cells to two *H. pylori* strains. Both Western blot and immunofluorescence assays indicated that after 24 h of *H. pylori* treatment, PIEZO1 and p‐p65 protein expression significantly rose (Figure 2I‐J). Similarly, in normal gastric epithelial cell line GSE1, Western blot results also demonstrated that the protein expression levels of PIEZO1 and p‐p65 significantly increased after 24 h of *H. pylori* treatment (Figure S3). Furthermore, the siRNA‐mediated knockdown of NFKB1 and RELA curtailed the PIEZO1 expression at the protein level in MKN28 and NCI‐N87 cells, reinforcing the notion that NF‐κB closely modulates PIEZO1 expression (Figure 2K,L). In the Hong Kong cohort, NFKB1/RELA and PIEZO1 expression in identical samples were assessed through IHC (Figure 2 M), and their protein levels showcased a direct correlation when scoring the positive cancer cell percentage (*n* = 128, Figure 2N,O). In the primary TGCA GC samples, NFKB1/RELA mRNA expression positively correlated with PIEZO1 expression (*n* = 375, Figure 2P–Q). These findings underscore that *H. pylori*‐induced GC stimulates NFKB1/RELA activation, which subsequently controls PIEZO1 expression.

### 3.3 Upregulated PIEZO1 activates YAP1 signaling to drive GC progression

**FIGURE 3:**
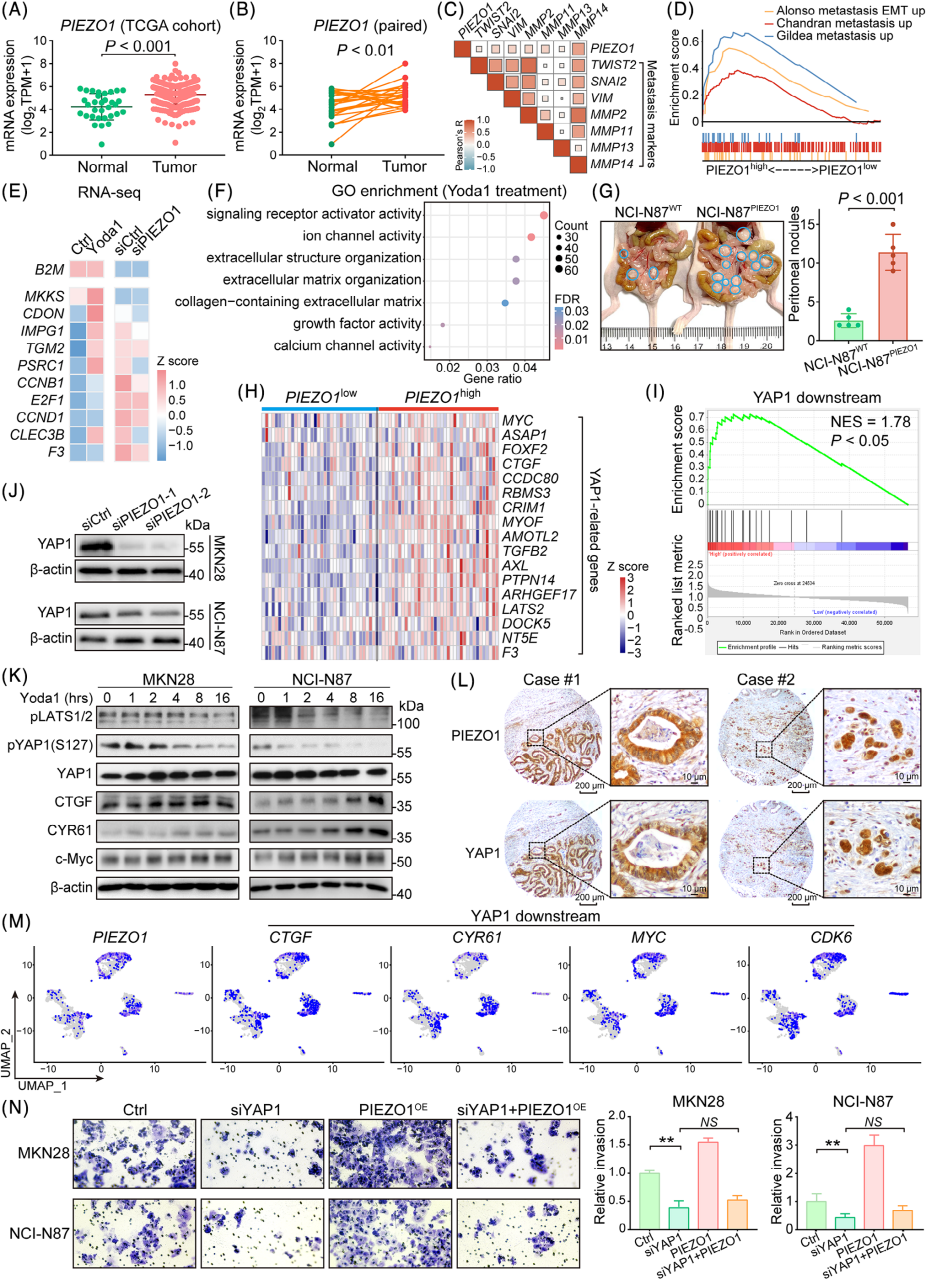
PIEZO1 exerts oncogenic functions in gastric cancer (GC) cancer cells and regulates the downstream Hippo pathway. (A) In TCGA cohorts, PIEZO1 mRNA expression was found to be elevated in tumour tissues compared to normal tissues. (B) PIEZO1 was notably up‐regulated in tumour samples when juxtaposed with their paired adjacent non‐tumour samples, highlighting its potential role in tumourigenesis. (C) Positive correlation was observed between the expression of PIEZO1 and metastasis‐associated genes in data extracted from TCGA. (D) GSEA demonstrated that high expression of PIEZO1 was positively correlated with tumour metastatic pathways. (E) Yoda1, an activator of PIEZO1, was found to escalate the expression of genes linked to both tumour metastasis and proliferation. Conversely, silencing PIEZO1 led to a suppression of these gene expressions. (F) GO enrichment bubble diagram demonstrating the pathway of DEG enrichment after Yoda1 intervention. (G) Overexpression of PIEZO1 in the NCI‐N87 cell line was found to spur GC peritoneal metastasis, implying its functional role in the metastatic process. (H) In the TCGA cohort, a high PIEZO1 expression group exhibited an activated YAP1 signature, indicating a possible regulatory relationship in GC samples. (I) GSEA results showed that elevated PIEZO1 expression positively correlates with the downstream pathways regulated by YAP1. (J) Knockdown of IEZO1 downregulated YAP1 expression in GC cells. (K) Yoda1 significantly induced Hippo pathway activation in a time‐dependent manner. (L) Protein expression of PIEZO1 and YAP1 was co‐localized in IHC‐stained samples from GC patients (scale bar, 200 μm for low magnification and 10 μm for high magnification). (M) Downstream genes of PIEZO1 and YAP1 were co‐expressed at the single‐cell level. (N) siYAP1 significantly suppressed the migration of GC cells, and its knockdown can entirely abolish the stimulatory effects of PIEZO1 overexpression.

To investigate the involvement of PIEZO1 in GC development more deeply, we delved into the TCGA dataset and observed an upregulation of PIEZO1 in tumour tissues (Figure 3A). Further analysis of paired GC samples confirmed this finding, showing a distinct upregulation of PIEZO1 in tumour samples relative to their paired normal tissue (*n* = 27, Figure 3B). Within the TCGA dataset, a positive correlation emerged between PIEZO1 expression and metastasis markers (Figure 3C). This correlation was further substantiated by GSEA, indicating a significant association of PIEZO1 with several metastasis markers including Alonso metastasis EMT up, Chandran metastasis up, and Gildea metastasis up (Figure 3D). Probing deeper into PIEZO1's role in GC, RNA‐seq experiments were conducted after stimulating PIEZO1 with Yoda1 (a PIEZO1 agonist) and subsequent PIEZO1 knockdown in NCI‐N87. Notably, Yoda1 stimulation bolstered the expression of metastasis and proliferation‐related genes, while PIEZO1 knockdown had the inverse effect (Figure 3E). GO analysis following Yoda1 stimulation further spotlighted PIEZO1's pivotal role as a signaling receptor in ion activity and ECM organization (Figure 3F; Table S12). As previously mentioned, the heightened expression of PIEZO1 can be attributed to the activation of the transcription factor NFKB1/RELA in both IM and tumour samples, implying an early onset of PIEZO1 up‐regulation. On a single‐cell level, comparison between the expression of PIEZO1 and IM goblet cell markers like *ATP2C2, ERN2* and *ACSS2* revealed a co‐localized high expression within the same cell population. This co‐expression hints at PIEZO1's potential role in the progression from IM to GC (Figure S6A). The escalated expression of PIEZO1 was tied to several processes including metastasis up‐regulation, cell migration, Myc target upregulation, and angiogenesis (Figure S6B–E). To corroborate the oncogenic function of PIEZO1, we carried out functional tests that illustrated that depleting PIEZO1 hindered patient‐derived organoid growth, curtailed monolayer colony formation of cancer cells, and compromised GC cell invasion (Figure S6F–H). Moreover, Western blot analyses showed that PIEZO1 depletion induced G1 cell cycle arrest and apoptosis, mirroring the effects of NFKB1/RELA knockdown (Figure S6I). To explore the role of PIEZO1 in GC metastasis, we developed a peritoneal metastasis model of GC using a highly metastatic NCI‐N87 cell line. Additionally, MKN28 and MKN45 are frequently employed to create models of GC metastasis through tail vein injection.^26^, ^27^ However, given our specific interest in investigating the mechanisms of CAFs involvement, we opted to utilize the peritoneal metastasis model. Overexpression of PIEZO1 further suggested its oncogenic role in GC progression by promoting peritoneal tumour dissemination (Figure 3G).

Building on our understanding of PIEZO1 as a mechanical sensor, and considering previous studies highlighting the activation of YAP1 due to increased cellular mechanical tension,^28^, ^29^ we endeavored to elucidate the relationship between PIEZO1 and downstream YAP1 signaling. Within the TCGA GC dataset, a high PIEZO1‐expression group exhibited a pronounced YAP1 signature, implying a potential functional link (Figure 3H). GSEA further identified a positive association between high PIEZO1 and YAP1 downstream (Figure 3I). Significantly, PIEZO1 knockdown led to a decline in YAP1 expression in both MKN28 and NCI‐N87 cells (Figure 3J). Given PIEZO1's role as a calcium channel protein, we assessed calcium influx in cancer cells. We found that while Yoda1 stimulation elevated Ca^2+^ influx, this effect was partially mitigated by GsMTx4 (a PIEZO1 antagonist) treatment (Figure S7A). In addition, the cancer cell lines MKN28 and NCI‐N87 showed a much greater degree of Ca^2+^ influx than the normal gastric epithelial cell line GSE1 (Figure S7B). Concurrently, Yoda1 treatment augmented YAP1 nuclear accumulation in NCI‐N87 cells, an effect that was neutralized by PIEZO1 depletion (Figure S7C‐D). Classic YAP1 signature genes such as CTGF, CYR61, MYC, and CDK6 showcased increased expression post Yoda1 stimulation, affirming the PIEZO1‐YAP1 signaling cascade (Figure 3K and S5C). IHC analysis drew attention to co‐expression of PIEZO1 and YAP1 within the same cancerous region, suggesting a strong correlation (Figure 3L). Further, GC scRNA‐seq analysis revealed a high *PIEZO1*‐expressing GC cell population, which also displayed elevated expression of CTGF, *CYR61*, *MYC* and *CDK6*. This highlighted the potential significance of the PIEZO1‐YAP1 axis in GC development (Figure 3 M). To determine the downstream significance of YAP1 in relation to PIEZO1, we conducted rescue experiments. These revealed that YAP1 knockdown nullified the stimulatory effects seen from PIEZO1 in a cell invasion assay (Figure 3N), reinforcing the crucial role of YAP1 as a downstream effector of PIEZO1 in GC.

### 3.4 The PIEZO1‐YAP1‐CTGF axis promotes CAF infiltration to remodel the tumour microenvironment in GC

**FIGURE 4:**
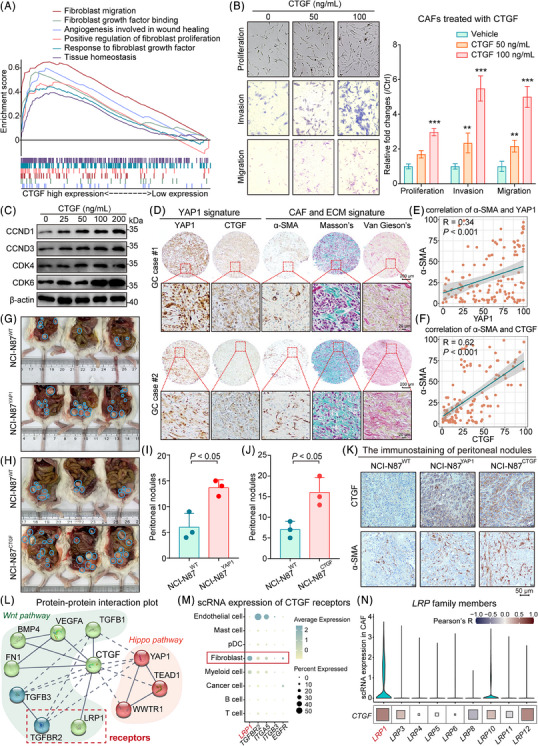
CTGF, a downstream gene of YAP1, promotes the viability of α‐SMA^+^ cancer‐associated fibroblasts (CAFs), thereby contributing to the gastric cancer (GC) progression. (A) GSEA indicated that elevated CTGF expression is positively associated with fibroblast migration and proliferation, suggesting its role in stromal remodeling within the tumour microenvironment. (B) CTGF was shown to significantly enhance the proliferative, migratory, and invasive capacities of CAFs in a dose‐dependent manner, reinforcing its role in tumour stroma dynamics. (C) CTGF upregulated the expression of proliferation‐related genes does‐dependently. (D–F) The protein levels of α‐SMA were positively correlated with PIEZO1 and CTGF in the original samples, respectively (scale bar, 200 μm for low magnification and 20 μm for high magnification). (G–J) Overexpression of YAP1 and CTGF in cancer cells significantly promoted GC peritoneal metastasis. (K) IHC of peritoneal nodes showed overexpression of YAP1 and CTGF, leading to an increase in α‐SMA. (L) Protein‐protein interaction plot indicated that CTGF is associated with the Wnt and Hippo pathways, of which TGFBR2 and LRP1 are receptors of CTGF. (M) scRNA‐seq analysis screening CTGF receptors revealed that fibroblast highly expresses LRP1 in GC. (N) Further single‐cell sequencing revealed that among the *LRP* family members, *LRP1* and *LRP10* were prominently expressed in fibroblasts. Moreover, a positive correlation between the expression levels of *CTGF* and *LRP1* was found in TCGA GC samples.

As a pivotal target of YAP1, CTGF exerts a crucial effect in mediating the oncogenic functions of the PIEZO1‐YAP1 axis. Analysis of the TCGA GC dataset revealed that elevated CTGF expression is significantly correlated with fibroblast activation, cellular proliferation, and migration, as demonstrated by GSEA (Figure 4A). Similarly, we observed that genes differentially expressed between GC patients with high and low CTGF expression are enriched in pathways related to the extracellular matrix (Figure S8).

Moreover, treatment of CAFs derived from primary GC samples with recombinant human CTGF markedly amplified their proliferative, invasive, and migratory capacities (Figure 4B). In alignment with these findings, CTGF stimulation led to an upregulation of cell cycle‐related proteins, including CCND1, CCND3, CDK4 and CDK6 (Figure 4C). Immunohistochemical analysis further corroborated a positive correlation between YAP1‐CTGF expression and α‐SMA^+^ CAF infiltration, as well as collagen fiber enrichment (Figure 4D‐F). Experimental overexpression of YAP1 or CTGF in NCI‐N87 cells notably increased the number of metastatic nodules in a peritoneal inoculation model (Figure 4G‐J), and immunostaining of these nodules confirmed an elevated presence of α‐SMA^+^ CAFs (Figure 4K). Protein‐protein interaction analyses revealed that CTGF is intricately linked to both the Wnt and Hippo signaling pathways, featuring interactions with key receptors TGFBR2 and LRP1 (Figure 4L). Intriguingly, single‐cell analysis indicated that LRP1 expression was most abundant in fibroblasts, and a positive correlation was observed between *CTGF* and *LRP1* expression levels in the TCGA dataset (Figure 4 M‐N). These observations preliminarily suggest that LRP1 may serve as a potential receptor for CTGF binding on α‐SMA^+^ CAFs.

### 3.5 PIEZO1 acts as a bridge between cancer cells and α‐SMA^+^ CAF to drive cancer progression in a positive feedback manner

**FIGURE 5:**
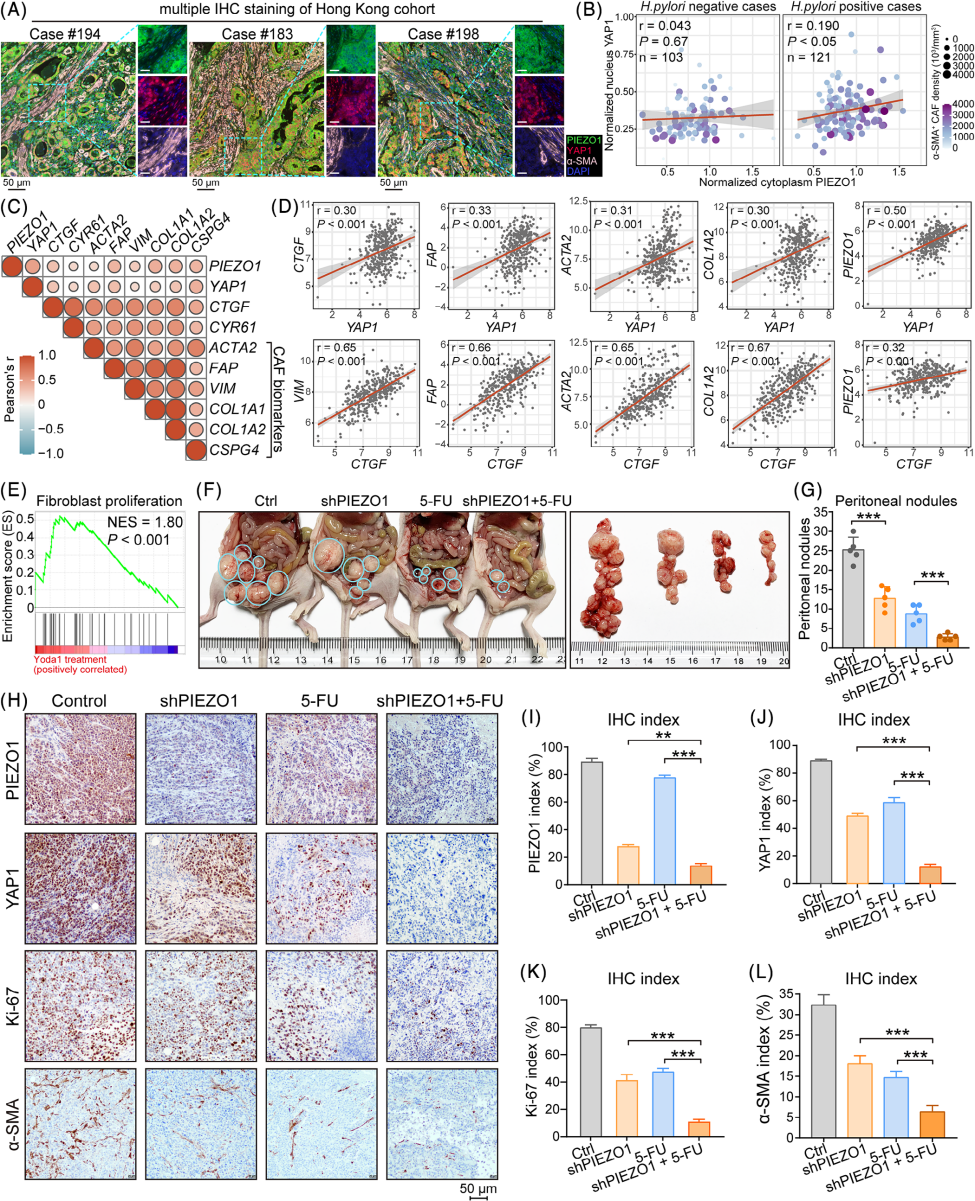
PIEZO1 acts as an essential bridge between cancer cells and α‐SMA^+^ cancer‐associated fibroblasts (CAFs) to regulate gastric cancer (GC) peritoneal metastasis. (A) Representative multiplex fluorescent immunohistochemical staining depicts the tumour microenvironment of *H. pylori* ^+^ and *H. pylori* ^−^ GC. PIEZO1 represents green, YAP1 represents red, α‐SMA represents pink, and DAPI represents blue (scale bar, 50 μm for low magnification and 20 μm for high magnification). (B) Correlation bubble plots of cytoplasmic PIEZO1 expression levels with cytosolic YAP1 expression levels in *H. pylori* ^+^ and *H. pylori* ^−^ GC, respectively. (C) The expression of PIEZO1 is positively correlated with the Hippo pathway and CAF biomarkers from TCGA cohort. (D) Detailed scatter plot of the correlation between CAF biomarkers and YAP1, CTGF, PIEZO1 (*n* = 375). (E) Regulation of fibroblast proliferation was positively correlated with Yoda1 treatment (*p* < .001). (F and G) PIEZO1 knockdown combined with 5‐FU achieved a synergistic effect in controlling tumour dissemination in a peritoneal metastasis model. (H) The representative IHC images of PIEZO1, YAP1, proliferation marker Ki‐67 and CAF marker α‐SMA in xenografts (scale bar, 50 μm). (I–L) Quantitative IHC indices of PIEZO1, YAP1, Ki‐67, and a‐SMA. **p < .01; ***p < .001.

To substantiate the close relationship between the PIEZO1‐YAP1 axis and α‐SMA^+^ CAF enrichment, we conducted multiplex immunohistochemical staining. The results clearly illustrated that α‐SMA^+^ CAFs were predominantly present in cases with elevated PIEZO1‐YAP1 expression (Figure 5A; Table S13). This positive correlation was particularly evident in *H. pylori^+^* GC cases, but was absent in *H. pylori^−^* cases, suggesting that *H. pylori* infection may act as a catalyst for PIEZO1‐YAP1 activation and subsequent CAF infiltration (Figure 5B). Furthermore, a correlation heatmap revealed that PIEZO1 expression was positively associated with *YAP1*, *CTGF*, and key CAF biomarkers such as *ACTA2*, *FAP*, and *COL1A1* (Figure 5C‐D). To reinforce the role of PIEZO1 in fibroblast‐driven cancer progression, we applied a fibroblast proliferation gene set to analyze Yoda1‐treated RNA‐seq data through GSEA, uncovering a positive association between PIEZO1 activation and fibroblast proliferation (Figure 5E).

In a clinical context, we assessed the potential impact of PIEZO1 targeting by treating PIEZO1‐knockdown cells with the first‐line anticancer drug 5‐FU. The combination of PIEZO1 knockdown and 5‐FU treatment exerted a synergistic effect in inhibiting tumour growth and dissemination in a peritoneal metastasis model (Figure 5F‐G). Subsequent immunohistochemical analysis of the xenografts indicated that this combinatorial approach significantly suppressed tumour growth and induced apoptosis, as evidenced by reduced Ki‐67 and α‐SMA expression (Figure 5H‐L). In vitro assays on MKN28 and NCI‐N87 cells showed that PIEZO1 knockdown substantially enhanced the growth‐inhibitory effects of 5‐FU, as corroborated by monolayer colony formation assays (Figure S9A). A similar synergistic effect was observed in patient‐derived organoid models (Figure S9B), pointing to an increased sensitivity of cancer cells to 5‐FU following PIEZO1 depletion.

In summary, our findings suggest that *H. pylori* infection accelerates early‐stage GC progression by upregulating NFKB1/RELA transcription mediated by PIEZO1 in cancer cells. We propose that the PIEZO1‐YAP1‐CTGF axis supports the proliferation of α‐SMA^+^ CAFs, which in turn may further stimulate PIEZO1 through collagen secretion, thereby establishing a self‐perpetuating loop that fuels GC progression.

### 3.6 CTGF predicts poor prognosis of GC and serves as a treatment target

**FIGURE 6:**
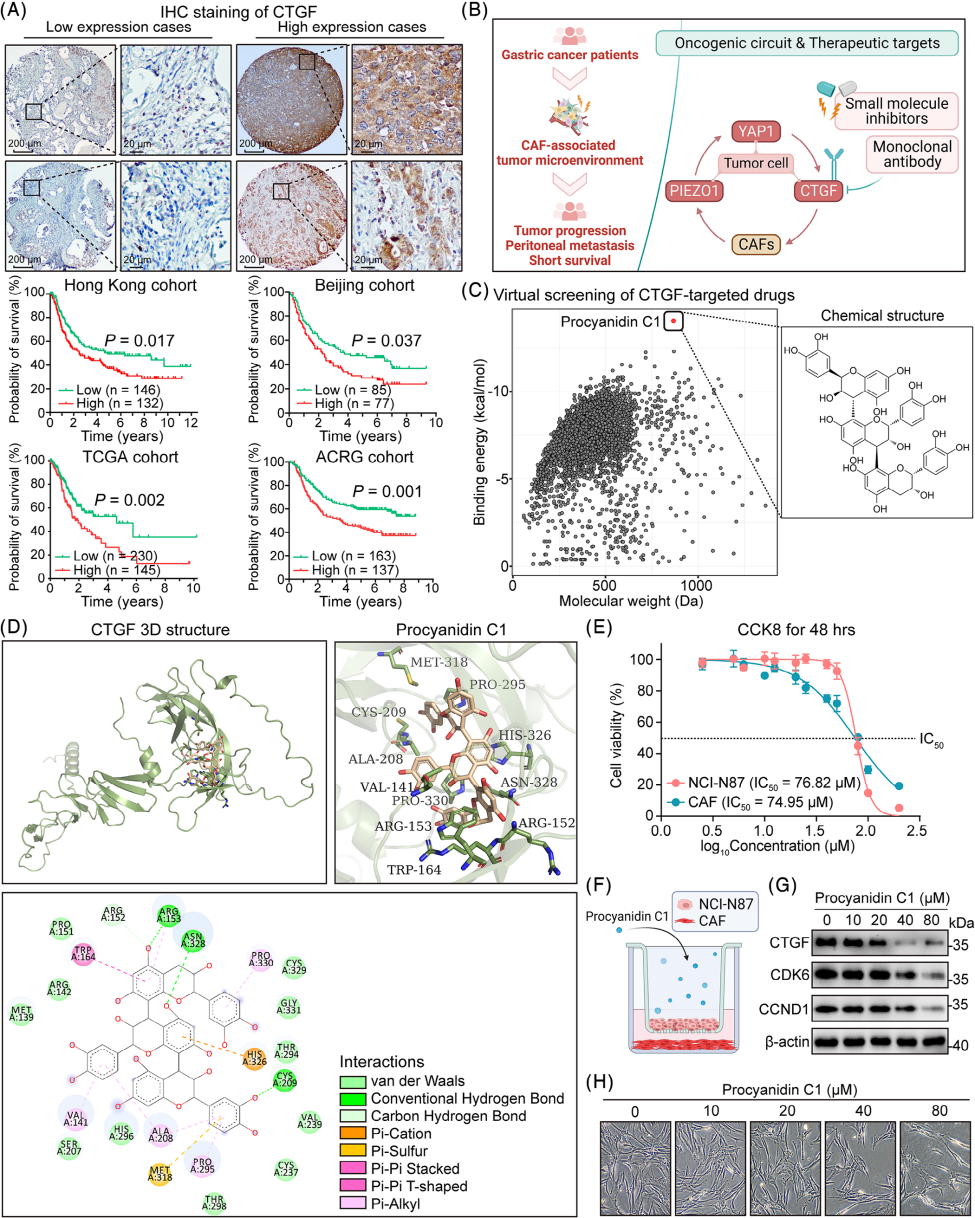
CTGF is a poor prognostic gene for gastric cancer (GC), and Procyanidin C1 is expected to be an effective CTGF inhibitor. (A) Representative images of IHC staining of CTGF in GC tissue microarrays from the Hong Kong cohort (scale bar, 200 μm for low magnification and 20 μm for high magnification). CTGF was mainly distributed in the cytoplasm and nearby extracellular matrix of cancer cells, while showing negative expression in the adjacent epithelial tissue. The high expression of CTGF was associated with unfavorable clinical outcomes (Hong Kong cohort, n = 278, *P* = .017; Beijing cohort, *n* = 162, *p* = .037; TCGA cohort, *n* = 375, *p* = .002; ACRG cohort, *n* = 300, *p* = .001). (B) Schematic diagram of the treatment strategy for GC patients. Blocking the interaction between tumour cells and cancer‐associated fibroblasts (CAFs) by inhibiting CTGF in the GC tumour microenvironment. (C) Scatter plot of computer‐aided drug screening using CTGF as a target (*n* = 4114, binding energy < 0). The top‐ranked Binding energy is Procyanidin C1. (D) The 3D structure of CTGF and Procyanidin C1 binding domain. (E) CCK‐8 examination of the viability of Procyanidin C1‐treated cancer cells and CAFs. (F) Schematic diagram of Procyanidin C1 treatment of NCI‐N87 and CAF co‐culture. (G) Procyanidin C1 inhibited CTGF and cell‐cycle‐related genes dose‐dependently. (H) Procyanidin C1 intervention with co‐cultured NCI‐N87 and CAF resulted in a dose‐dependent inhibition of CAF growth.

As a key driver of CAFs in GC, elevated levels of CTGF are strongly correlated with poor patient outcomes across multiple GC datasets (Figure 6A). This increased CTGF expression is primarily localized in the cytoplasm of the cancer cells. Our prior research established a self‐reinforcing loop involving the co‐activation of PIEZO1, YAP1‐CTGF and CAFs, which collectively fuels GC progression (Figure 6B). With CTGF emerging as a viable therapeutic target to break this cycle, we conducted molecular docking studies to identify potent small molecules targeting CTGF. Notably, Procyanidin C1 was identified as the most effective candidate from a library of 4511 anti‐cancer compounds (Figure 6C). The 3D structure of CTGF and its drug‐protein binding sites were also predicted (Figure 6D). To evaluate Procyanidin C1's therapeutic efficacy, we assessed its 48‐h IC_50_ in both NCI‐N87 cells and CAFs and found that CAFs were notably more sensitive to this compound (Figure 6E). Subsequent co‐culture experiments involving NCI‐N87 cells and CAFs, treated with Procyanidin C1, revealed a dose‐dependent reduction in CTGF and cell‐cycle proteins like CDK6 and CCND1 within just 24 h of treatment (Figure 6F,G). Moreover, microscopic analysis confirmed a significant reduction in CAF numbers at increasing Procyanidin C1 concentrations; remarkably, a 40 μM dose resulted in roughly a 50% reduction in CAF numbers after only 24 h, underscoring its ability to suppress CAF proliferation primarily by downregulating CTGF expression in cancer cells (Figure 6H). In conclusion, our findings strongly suggest that Procyanidin C1 holds significant therapeutic promise by effectively inhibiting CTGF expression and arresting CAF proliferation in a co‐culture system of NCI‐N87 cells and CAFs.

### 3.7 Targeting CTGF is an effective strategy to inhibit GC progression and metastasis

**FIGURE 7:**
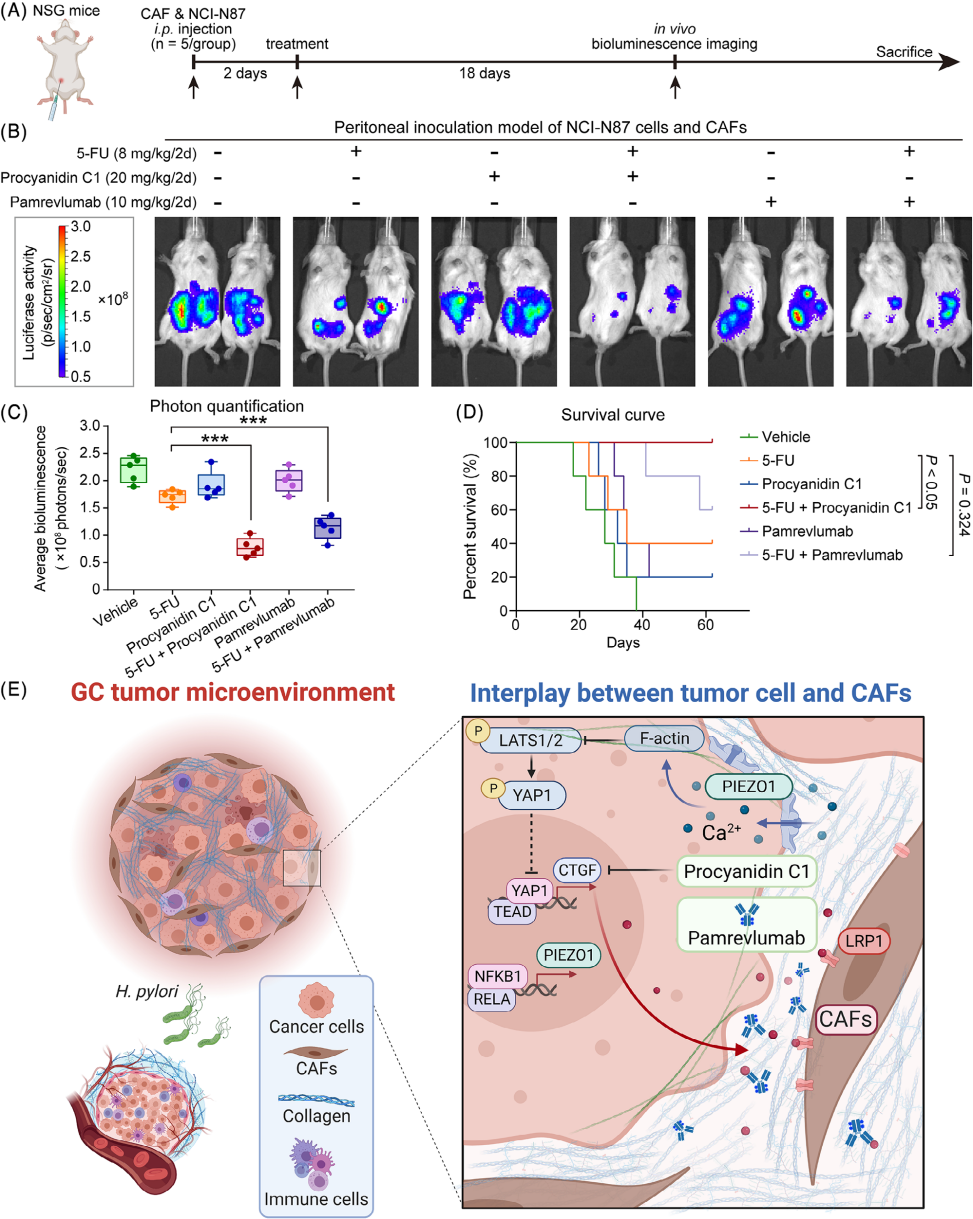
Targeting CTGF synergizes with 5‐FU to arrest peritoneal metastasis of gastric cancer (GC). (A) Schematic diagram of the in vivo experiment. (B) Photographs of peritoneal metastases in the vehicle group, 5‐FU group, Procyanidin C1 group, Pamrevlumab group, and co‐drug groups. (C) Photon quantification of GC peritoneal metastasis in NSG mice inoculated with NCI‐N87 cells and cancer‐associated fibroblasts (CAFs). (D) Survival curves of NSG mice in each intervention group. (E) The overall schematic presentation of the project. Based on the molecular features of cancer cells and enrichment of CAFs in GC, blocking the CTGF effectively disrupts the mutual remodeling of cancer cells and CAFs, inhibits the GC peritoneal metastasis, and serves as a potential novel therapeutic strategy to prolong GC patient survival.

To rigorously assess the impact of CTGF targeting in GC metastasis, we employed a peritoneal metastasis NSG mouse model for in vivo experimentation. Figure 7A illustrates the intraperitoneal injection protocol, using NCI‐N87 cell line in conjunction with CAFs. Remarkably, the combination of 5‐FU with the CTGF‐specific small molecule Procyanidin C1 displayed a synergistic effect in mitigating GC peritoneal metastasis after 14 days of treatment, a result parallel to that of Pamrevlumab, a humanized monoclonal antibody targeting CTGF (Figure 7B,C). Moreover, survival curves indicated a statistically significant improvement in prognosis for the group receiving Procyanidin C1 in combination with 5‐FU, as compared to the 5‐FU monotherapy group (*p* < .05). Intriguingly, the combination of Pamrevlumab and 5‐FU did not yield a statistically significant extension in mouse survival (Figure 7D).

In summary, *H. pylori*‐induced inflammation in early‐stage GC amplifies the NFKB1/RELA transcriptional activity on *PIEZO1*, thereby facilitating GC progression. The resultant activation of the PIEZO1‐YAP1‐CTGF signaling axis in GC cells encourages the recruitment of α‐SMA^+^ CAFs, further activating PIEZO1 and creating a self‐sustaining loop. Pharmacologically inhibiting CTGF effectively dismantles this mutualistic interaction between cancer cells and α‐SMA^+^ CAFs, thereby suppressing peritoneal metastasis and enhancing patient survival rates. Our findings strongly advocate for CTGF inhibition as a novel adjuvant therapeutic strategy, particularly when paired with 5‐FU, for GC patients with a high prevalence of CAFs (Figure 7E).

## 4 DISCUSSION

In this study, we elucidated the role of *H. pylori*‐induced inflammation in activating the NF‐κB signaling pathway in GC cells. This activation subsequently triggers the PIEZO1‐YAP1‐CTGF axis, leading to the secretion of CTGF, which promotes the viability of α‐SMA^+^ CAFs. This results in an accumulation of α‐SMA^+^ CAFs, contributing to the stiffening of the tumour microenvironment. Such potential stiffening further activates PIEZO1, creating a self‐perpetuating cycle. Notably, we demonstrate that this positive feedback loop can be effectively disrupted by targeting CTGF, as summarized in Figure 7E.

The World Health Organization classifies *H. pylori* as a Class I carcinogen, attributing its chronic infection as the most significant risk factor for gastric adenocarcinoma. It is reported that 90% of all non‐cardia GCs are associated with *H. pylori* infection.^30^ Variability among *H. pylori* strains plays a pivotal role in GC pathogenesis; for instance, patients infected with CagA‐positive strains have a higher propensity to develop GC.^31^ Importantly, we observed a high concentration of α‐SMA^+^ CAFs in *H. pylori* ^+^ GC tissues and found that elevated levels of α‐SMA were associated with poor clinical outcomes (Figure 1). Previous studies have indicated that *H. pylori* induces the expression of pro‐inflammatory cytokines and activates the NF‐κB signaling cascade in gastric epithelial cells.^32^, ^33^ Additionally, *H. pylori*‐induced degradation of IκBα amplifies NF‐κB nuclear accumulation in GC.^34^ Our prior results demonstrated that the key components of the canonical NF‐κB signaling pathway, NFKB1 and RELA, were significantly upregulated at the protein level in GC and played a crucial role in the transition from inflammation to cancer.^21^ Given these findings, there is an urgent imperative to identify novel downstream effectors of the NF‐κB pathway to better understand its oncogenic role and its association with α‐SMA^+^ CAFs.

In this study, we identified PIEZO1 as a critical target of NF‐κB signaling. Notably, data from both the TCGA and Hong Kong cohorts demonstrated a positive correlation between NF‐κB and PIEZO1. In cases positive for *H. pylori* infection, NF‐κB signaling was actively engaged, leading to an up‐regulation of PIEZO1 expression. It is worth mentioning that aside from NF‐κB, YAP1 has also been reported to regulate PIEZO1 expression.^35^ As a mechanosensitive ion channel protein, PIEZO1 is predominantly expressed in tissues like endothelial cells, bladder, kidney and lung epithelial cells where mechanosensation plays critical roles.^36^, ^37^ Intriguingly, studies have indicated that PIEZO1 can enhance aggressiveness in glioma by modulating tissue mechanics.^38^ Additionally, PIEZO1 is activated to maintain epithelial monolayer homeostasis in situations of cell crowding and cytoskeletal contractions.^39^ In our IHC‐stained sections, we observed that PIEZO1 was primarily expressed in cancer cells (Figure 3L). Moreover, overexpression of PIEZO1 led to enhanced peritoneal metastasis of GC cells in nude mice (Figure 3G). Based on these findings, we firmly conclude that PIEZO1 has an oncogenic effect in GC.

Pivoting to the functional aspects of PIEZO1 as a mechanosensor in the microenvironment of GC, we sought to explore how PIEZO1 mediates downstream signaling in cancer cells. Analysis of the TCGA dataset revealed upregulation of YAP1‐related genes in tissue samples from patients with high PIEZO1 expression (Figure 3I). This was corroborated by GSEA results, which showed that cases with high levels of PIEZO1 exhibited an activated YAP1 signature (Figure 3H). Previous research has identified YAP1 as a driver oncoprotein in GC, and its elevated expression has been linked to poor clinical outcomes in GC patients.^40^, ^41^ Given the oncogenic role of YAP1 in GC, molecular mechanisms involving YAP1 have received considerable attention.^42^, ^43^ YAP1 predominantly interacts with TEAD transcription factors to promote gastric tumourigenesis.^44^, ^45^ As a transcriptional co‐activator, YAP1 directly upregulates the expression of CTGF, CYR61, c‐Myc, and CDK6.^17^, ^46^ The PIEZO1 agonist, Yoda1, activates YAP1 signature genes in a time‐dependent manner. Through Ca^2+^ influx, Yoda1 indirectly inhibits LATS1/2 phosphorylation, leading to the dephosphorylation of LATS1/2 and the removal of its inhibitory effect on YAP1. This results in the nuclear accumulation of YAP1 and an increase in CTGF expression. At the single‐cell resolution level, PIEZO1 and YAP1 signature genes co‐expressed in the same cell populations, indicating a tight regulatory relationship between PIEZO1 and YAP1. Therefore, we confirm that PIEZO1 promotes GC progression primarily through the activation of the YAP1 signature, which is consistent with studies on cholangiocarcinoma.^47^

Reflecting upon our earlier findings, we observed a pronounced presence of α‐SMA^+^ CAFs in *H. pylori* ^+^ GC tissue sections. CAFs, a predominant cell population within stromal cells, have long been recognized for their association with poor patient prognosis.^48^ A plethora of research has portrayed CAFs as prolific producers of growth factors, cytokines, chemokines, metabolites and ECM.^49^ Intriguingly, in our research, CTGF, a downstream effector of YAP1, was identified as a key factor driving the accumulation and aggressiveness of CAFs (Figure 4). CTGF's pivotal role in spearheading fibrotic diseases and its involvement in various cancers has been well‐documented.^50^ Elevated CTGF levels within CAFs have been associated with a reduced disease‐free survival rate in patients.^51^, ^52^, ^53^, ^54^ Remarkably, our co‐staining experiments involving α‐SMA, PIEZO1 and YAP1 unveiled a positive interrelation among these markers in *H. pylori* ^+^ GC patients (Figure 5A,B). This observation was further substantiated by the TCGA GC database, which highlighted a positive correlation between YAP1‐CTGF, PIEZO1 and CAF biomarkers (Figure 5C,D). In a parallel context, α‐SMA expression, indicative of hepatic stellate cell activation, has been shown to correlate positively with CTGF expression, thereby accelerating the progression of hepatocellular carcinoma.^55^ Furthermore, a prevailing hypothesis regarding the pro‐fibrotic effects of CTGF posits that it facilitates the presentation of TGF‐β to its respective receptor, thereby playing a crucial role in TGF‐β signaling pathways.^56^, ^57^ Current clinical trials aiming to mitigate pulmonary fibrosis are exploring the use of antibodies designed to inhibit the interaction between TGF‐β and CTGF.^58^ Notably, the fourth domain of CTGF, along with the WNT signaling pathway, represents promising novel therapeutic targets in fibrotic diseases.^59^ Our protein‐protein interaction plot (Figure 4L) similarly substantiates that CTGF serves as a crucial intermediary, facilitating crosstalk between the Hippo and Wnt signaling pathways. Additionally, CTGF engages in interactions with two receptors, LRP1 and TGFBR2, positioning it as a compelling target for therapeutic intervention. Collectively, these insights cement the existence of a PIEZO1‐YAP1‐CTGF axis in cancer cells, which, under the regulation of NFκB signaling, potentiates the proliferation of α‐SMA^+^ CAFs.

Equally critical to highlight is the role of the ECM, primarily synthesized by α‐SMA+ CAFs, which serves as a hallmark in the tumour microenvironment.^48^, ^60^ The potential rigidity of the ECM, which is based on known consequences of collagen accumulation and CTGF activity and their established roles in other contexts, has been positively associated with the malignancy status across various solid tumours.^61^, ^62^, ^63^ Within epithelial cells, an increase in potential ECM rigidity can drive epithelial‐mesenchymal transition (EMT), facilitated by YAP1 activation via mechanotransduction pathways. This activated YAP1, in turn, augments the expression of metalloproteinases, amplifying the stiffness of the ECM.^64^ This dynamic interplay between YAP1 and the ECM composition not only perpetuates tumour growth and metastasis but also poses resistance to chemotherapy.^65^, ^66^ Our study pioneers in elucidating how the mechanosensor PIEZO1, potentially responsive to collagen stiffness, mediates the activation of YAP1‐CTGF through Ca^2+^ influx, thereby bridging a knowledge gap and offering an explanation as to why YAP1 has also been categorized as a mechanosensor.

Given the elevated expression of the PIEZO1‐YAP1‐CTGF axis, which serves as a linchpin in tumour development and encourages peritoneal metastasis, therapeutically targeting this oncogenic pathway could effectively halt the progression from IM to GC. Particularly compelling is the role of CTGF, which serves as a crucial mediator in the intercellular crosstalk between cancer cells and CAFs, thereby positioning it as an attractive therapeutic target. Various molecular entities, ranging from antibodies and siRNAs to short hairpin RNAs (shRNAs) and natural compounds, have been engineered to target CTGF in cancer research. For instance, Pamrevlumab (also known as FG‐3019), a humanized anti‐CTGF antibody, has undergone extensive clinical trials for conditions such as muscular dystrophy, liver fibrosis, idiopathic pulmonary fibrosis and pancreatic cancer.^67^, ^68^ Remarkably, our study introduces Procyanidin C1, a novel small molecule that specifically targets CTGF. Comparative analyses using GC peritoneal metastasis models affirm that Procyanidin C1 amplifies the efficacy of 5‐Fluorouracil (5‐FU) more robustly than Pamrevlumab does (Figures 6 and 7).

## 5 CONCLUSIONS

In summary, the mechanosensor PIEZO1, which is abundantly expressed in GC, is directly modulated by NF‐κB signalling. PIEZO1 augments the progression from IM to GC primarily through the activation of the YAP1‐CTGF pathway. The CTGF secreted from cancer cells may orchestrate a CAF‐enriched microenvironment, thereby initiating a self‐perpetuating positive feedback loop via PIEZO1 activation. Therefore, targeting either PIEZO1 or CTGF could represent a viable strategy for obstructing tumour progression and peritoneal metastasis in GC patients. Our findings not only deepen our comprehension of the mechanistic underpinnings that drive the early onset of GC but also introduce novel therapeutic avenues to extend the survival of patients afflicted with this malignancy.

## AUTHOR CONTRIBUTIONS

WK designed the study. BC and XL conducted most of the experiments, interpreted the results, and drafted the manuscript. PY, WNC, CF, JZ, AHKC, and CC contributed to the acquisition of clinical samples. BC, GWML, SS, KF and FX contributed to the in vivo studies. JSHK, KTL, BZ, SW, and DX contributed to the bioinformatic analysis. CCW, WKKW, MWYC, PMKT, CMT, KWL, GMKT, JY, and KFT gave professional comments on the study and revised the manuscript. All authors have approved the final manuscript.

## CONFLICT OF INTEREST STATEMENT

The authors declare that they have no competing interests.

## ETHICAL APPROVAL

The related GC study protocols were approved by the Animal Experimentation Ethics Committee (AEEC) and The Joint Chinese University of Hong Kong‐New Territories East Cluster Clinical Research Ethics Committee (The Joint CUHK‐NTEC CREC).

## Supporting information

Supporting Information

## Data Availability

The datasets analyzed during this study are available from the corresponding author upon reasonable request.

## References

[1] Sung H , Ferlay J , Siegel RL , et al. Global cancer statistics 2020: GLOBOCAN estimates of incidence and mortality worldwide for 36 cancers in 185 countries. CA Cancer J Clin. 2009;71(3):209‐249. doi:10.3322/caac.21660 33538338

[2] Gao L , Michel A , Weck MN , et al. Helicobacter pylori infection and gastric cancer risk: evaluation of 15 H. pylori proteins determined by novel multiplex serology. Cancer Res. 2009;69:6164‐6170. doi:10.1158/0008-5472.Can-09-0596 19602590

[3] Wang F , Meng W , Wang B , Qiao L . Helicobacter pylori‐induced gastric inflammation and gastric cancer. Cancer Lett. 2014;345:196‐202. doi:10.1016/j.canlet.2013.08.016 23981572

[4] Gupta S , Li D , El Serag HB , et al. AGA clinical practice guidelines on management of gastric intestinal metaplasia. Gastroenterology. 2020;158:693‐702. doi:10.1053/j.gastro.2019.12.003 31816298 PMC7340330

[5] Shah SC , Gupta S , Li D , et al. Spotlight: gastric intestinal metaplasia. Gastroenterology. 2020;158:704. doi:10.1053/j.gastro.2020.01.012 32035529

[6] Huang KK , Ramnarayanan K , Zhu F , et al. Genomic and epigenomic profiling of high‐risk intestinal metaplasia reveals molecular determinants of progression to gastric cancer. Cancer Cell. 2018;33:137‐150. doi:10.1016/j.ccell.2017.11.018 29290541

[7] Ford AC , Yuan Y , Moayyedi P . Helicobacter pylori eradication therapy to prevent gastric cancer: systematic review and meta‐analysis. Gut. 2020;69:2113‐2121. doi:10.1136/gutjnl-2020-320839 32205420

[8] Zhang J , Zhou Y , Huang T , et al. PIEZO1 functions as a potential oncogene by promoting cell proliferation and migration in gastric carcinogenesis. Mol Carcinog. 2018;57:1144‐1155. doi:10.1002/mc.22831 29683214

[9] Yang XN , Lu Y‐P , Liu J‐J , et al. Piezo1 is as a novel trefoil factor family 1 binding protein that promotes gastric cancer cell mobility in vitro. Dig Dis Sci. 2014;59:1428‐1435. doi:10.1007/s10620-014-3044-3 24798994

[10] Wang X , Cheng G , Miao Y , et al. Piezo type mechanosensitive ion channel component 1 facilitates gastric cancer omentum metastasis. J Cell Mol Med. 2021;25:2238‐2253. doi:10.1111/jcmm.16217 33439514 PMC7882944

[11] Lin YC , Guo YR , Miyagi A , et al. Force‐induced conformational changes in PIEZO1. Nature. 2019;573:230‐234. doi:10.1038/s41586-019-1499-2 31435018 PMC7258172

[12] Benn MC , Pot SA , Moeller J , et al. How the mechanobiology orchestrates the iterative and reciprocal ECM‐cell cross‐talk that drives microtissue growth. Sci Adv. 2023;9:eadd9275. doi:10.1126/sciadv.add9275 36989370 PMC10058249

[13] Hanley CJ , Mellone M , Ford K , et al. Targeting the myofibroblastic cancer‐associated fibroblast phenotype through inhibition of NOX4. J Natl Cancer Inst. 2018;110:109‐120. doi:10.1093/jnci/djx121 28922779 PMC5903651

[14] Leung KT , Zhang C , Chan KYY , et al. CD9 blockade suppresses disease progression of high‐risk pediatric B‐cell precursor acute lymphoblastic leukemia and enhances chemosensitivity. Leukemia. 2020;34:709‐720. doi:10.1038/s41375-019-0593-7 31624373

[15] Huch M , Gehart H , van Boxtel R , et al. Long‐term culture of genome‐stable bipotent stem cells from adult human liver. Cell. 2015;160:299‐312. doi:10.1016/j.cell.2014.11.050 25533785 PMC4313365

[16] Chen B , Song Y , Zhan Y , et al. Fangchinoline inhibits non‐small cell lung cancer metastasis by reversing epithelial‐mesenchymal transition and suppressing the cytosolic ROS‐related Akt‐mTOR signaling pathway. Cancer Lett. 2022;543:215783. doi:10.1016/j.canlet.2022.215783 35700820

[17] Kang W , Huang T , Zhou Y , et al. miR‐375 is involved in Hippo pathway by targeting YAP1/TEAD4‐CTGF axis in gastric carcinogenesis. Cell Death Dis. 2018;9:92. doi:10.1038/s41419-017-0134-0 29367737 PMC5833783

[18] Sathe A , Grimes SM , Lau BT , et al. Single‐cell genomic characterization reveals the cellular reprogramming of the gastric tumor microenvironment. Clin Cancer Res. 2020;26:2640‐2653. doi:10.1158/1078-0432.CCR-19-3231 32060101 PMC7269843

[19] Trapnell C , Cacchiarelli D , Grimsby J , et al. The dynamics and regulators of cell fate decisions are revealed by pseudotemporal ordering of single cells. Nat Biotechnol. 2014;32:381‐386. doi:10.1038/nbt.2859 24658644 PMC4122333

[20] Trapnell C , Roberts A , Goff L , et al. Differential gene and transcript expression analysis of RNA‐seq experiments with TopHat and Cufflinks. Nat Protoc. 2012;7:562‐578. doi:10.1038/nprot.2012.016 22383036 PMC3334321

[21] Huang T , Kang W , Zhang B , et al. miR‐508‐3p concordantly silences NFKB1 and RELA to inactivate canonical NF‐kappaB signaling in gastric carcinogenesis. Mol Cancer. 2016;15:9. doi:10.1186/s12943-016-0493-7 26801246 PMC4724081

[22] Jumper J , Evans R , Pritzel A , et al. Highly accurate protein structure prediction with AlphaFold. Nature. 2021;596:583‐589. doi:10.1038/s41586-021-03819-2 34265844 PMC8371605

[23] Kim S , Thiessen PA , Bolton EE , et al. PubChem substance and compound databases. Nucleic Acids Res. 2016;44:D1202‐D1213. doi:10.1093/nar/gkv951 26400175 PMC4702940

[24] Jendele L , Krivak R , Skoda P , Novotny M , Hoksza D . PrankWeb: a web server for ligand binding site prediction and visualization. Nucleic Acids Res. 2019;47:W345‐W349. doi:10.1093/nar/gkz424 31114880 PMC6602436

[25] Morris GM , Huey R , Lindstrom W , et al. AutoDock4 and AutoDockTools4: automated docking with selective receptor flexibility. J Comput Chem. 2009;30:2785‐2791. doi:10.1002/jcc.21256 19399780 PMC2760638

[26] Wang X , Ye T , Xue B , et al. Mitochondrial GRIM‐19 deficiency facilitates gastric cancer metastasis through oncogenic ROS‐NRF2‐HO‐1 axis via a NRF2‐HO‐1 loop. Gastric Cancer. 2021;24:117‐132. doi:10.1007/s10120-020-01111-2 32770429

[27] Ye T , Yang M , Huang D , et al. MicroRNA‐7 as a potential therapeutic target for aberrant NF‐κB‐driven distant metastasis of gastric cancer. J Exp Clin Cancer Res. 2019;38:55. doi:10.1186/s13046-019-1074-6 30728051 PMC6364399

[28] Zhao Q , Zhou H , Chi S , et al. Structure and mechanogating mechanism of the Piezo1 channel. Nature. 2018;554:487‐492. doi:10.1038/nature25743 29469092

[29] Zhang J , Zhou Y , Tang P , et al. Mechanotransduction and cytoskeleton remodeling shaping YAP1 in gastric tumorigenesis. Int J Mol Sci. 2019;20(7):1576. doi:10.3390/ijms20071576 30934860 PMC6480114

[30] Arnold M , Ferlay J , van Berge Henegouwen MI , Soerjomataram I . Global burden of oesophageal and gastric cancer by histology and subsite in 2018. Gut. 2020;69:1564‐1571. doi:10.1136/gutjnl-2020-321600 32606208

[31] Zhao Y , Zhang J , Cheng ASL , et al. Gastric cancer: genome damaged by bugs. Oncogene. 2020;39:3427‐3442. doi:10.1038/s41388-020-1241-4 32123313 PMC7176583

[32] Cao L , Zhu S , Lu H , et al. Helicobacter pylori‐induced RASAL2 through activation of nuclear factor‐κB promotes gastric tumorigenesis via β‐catenin signaling axis. Gastroenterology. 2022;162:1716‐1731. doi:10.1053/j.gastro.2022.01.046 35134322 PMC9038683

[33] Zabaleta J . Multifactorial etiology of gastric cancer. Methods Mol Biol. 2012;863:411‐435. doi:10.1007/978-1-61779-612-8_26 22359309 PMC3625139

[34] Maeda S , Yoshida H , Ogura K , et al. H. pylori activates NF‐kappaB through a signaling pathway involving IkappaB kinases, NF‐kappaB‐inducing kinase, TRAF2, and TRAF6 in gastric cancer cells. Gastroenterology. 2000;119:97‐108. doi:10.1053/gast.2000.8540 10889159

[35] Hasegawa K , Fujii S , Matsumoto S , et al. YAP signaling induces PIEZO1 to promote oral squamous cell carcinoma cell proliferation. J Pathol. 2021;253:80‐93. doi:10.1002/path.5553 32985688

[36] Bagriantsev SN , Gracheva EO , Gallagher PG . Piezo proteins: regulators of mechanosensation and other cellular processes. J Biol Chem. 2014;289:31673‐31681. doi:10.1074/jbc.R114.612697 25305018 PMC4231648

[37] Wang S , Wang B , Shi Y , et al. Mechanosensation by endothelial PIEZO1 is required for leukocyte diapedesis. Blood. 2022;140:171‐183. doi:10.1182/blood.2021014614 35443048 PMC9305087

[38] Chen X , Wanggou S , Bodalia A , et al. A feedforward mechanism mediated by mechanosensitive ion channel PIEZO1 and tissue mechanics promotes glioma aggression. Neuron. 2018;100:799‐815. doi:10.1016/j.neuron.2018.09.046 30344046

[39] Gudipaty SA , Lindblom J , Loftus PD , et al. Mechanical stretch triggers rapid epithelial cell division through Piezo1. Nature. 2017;543:118‐121. doi:10.1038/nature21407 28199303 PMC5334365

[40] Kang W , Tong JHM , Chan AWH , et al. Yes‐associated protein 1 exhibits oncogenic property in gastric cancer and its nuclear accumulation associates with poor prognosis. Clin Cancer Res. 2011;17:2130‐2139. doi:10.1158/1078-0432.CCR-10-2467 21346147

[41] Ajani JA , Xu Y , Huo L , et al. YAP1 mediates gastric adenocarcinoma peritoneal metastases that are attenuated by YAP1 inhibition. Gut. 2021;70:55‐66. doi:10.1136/gutjnl-2019-319748 32345613 PMC9832914

[42] Kang W , Cheng AS , Yu J , To KF . Emerging role of Hippo pathway in gastric and other gastrointestinal cancers. World J Gastroenterol. 2016;22:1279‐1288. doi:10.3748/wjg.v22.i3.1279 26811664 PMC4716037

[43] Yao H , Ren D , Wang Y , et al. KCTD9 inhibits the Wnt/β‐catenin pathway by decreasing the level of β‐catenin in colorectal cancer. Cell Death Dis. 2022;13:761. doi:10.1038/s41419-022-05200-1 36055981 PMC9440223

[44] Zhou Y , Huang T , Cheng A , et al. The TEAD family and its oncogenic role in promoting tumorigenesis. Int J Mol Sci. 2016;17(1):138. doi:10.3390/ijms17010138 26805820 PMC4730377

[45] Zhou Y , Huang T , Zhang J , et al. TEAD1/4 exerts oncogenic role and is negatively regulated by miR‐4269 in gastric tumorigenesis. Oncogene. 2017;36(47):6518‐6530. 28759040 10.1038/onc.2017.257 PMC5702719

[46] Choi W , Kim J , Park J , et al. YAP/TAZ initiates gastric tumorigenesis via upregulation of MYC. Cancer Res. 2018;78:3306‐3320. doi:10.1158/0008-5472.Can-17-3487 29669762

[47] Zhu B , Qian W , Han C , Bai T , Hou X . Piezo 1 activation facilitates cholangiocarcinoma metastasis via Hippo/YAP signaling axis. Mol Ther Nucleic Acids. 2021;24:241‐252. doi:10.1016/j.omtn.2021.02.026 33767919 PMC7973248

[48] Caligiuri G , Tuveson DA . Activated fibroblasts in cancer: perspectives and challenges. Cancer Cell. 2023;41:434‐449. doi:10.1016/j.ccell.2023.02.015 36917949 PMC11022589

[49] Yamamura Y , Asai N , Enomoto A , et al. Akt‐Girdin signaling in cancer‐associated fibroblasts contributes to tumor progression. Cancer Res. 2015;75:813‐823. doi:10.1158/0008-5472.CAN-14-1317 25732845

[50] Wang MY , Chen P‐S , Prakash E , et al. Connective tissue growth factor confers drug resistance in breast cancer through concomitant up‐regulation of Bcl‐xL and cIAP1. Cancer Res. 2009;69:3482‐3491. doi:10.1158/0008-5472.CAN-08-2524 19351859

[51] Hutchenreuther J , Vincent K , Norley C , et al. Activation of cancer‐associated fibroblasts is required for tumor neovascularization in a murine model of melanoma. Matrix Biol. 2018;74:52‐61. doi:10.1016/j.matbio.2018.06.003 29885461

[52] Ma S , Kanai R , Pobbati AV , et al. The TAZ‐CAMTA1 fusion protein promotes tumorigenesis via connective tissue growth factor and Ras‐MAPK signaling in epithelioid hemangioendothelioma. Clin Cancer Res. 2022;28:3116‐3126. doi:10.1158/1078-0432.Ccr-22-0421 35443056 PMC9306355

[53] Shephard AP , Giles P , Mbengue M , et al. Stroma‐derived extracellular vesicle mRNA signatures inform histological nature of prostate cancer. J Extracell Vesicles. 2021;10:e12150. doi:10.1002/jev2.12150 34596356 PMC8485336

[54] Shimo T , Yoshioka N , Takigawa M , Sasaki A . Analysis of pathological activities of CCN proteins in bone metastasis. Methods Mol Biol. 2017;1489:505‐512. doi:10.1007/978-1-4939-6430-7_42 27734401

[55] Makino Y , Hikita H , Kodama T , et al. CTGF mediates tumor‐stroma interactions between hepatoma cells and hepatic stellate cells to accelerate HCC progression. Cancer Res. 2018;78:4902‐4914. doi:10.1158/0008-5472.Can-17-3844 29967264

[56] Grotendorst GR . Connective tissue growth factor: a mediator of TGF‐beta action on fibroblasts. Cytokine Growth Factor Rev. 1997;8:171‐179. doi:10.1016/s1359-6101(97)00010-5 9462483

[57] Leask A , Denton CP , Abraham DJ . Insights into the molecular mechanism of chronic fibrosis: the role of connective tissue growth factor in scleroderma. J Invest Dermatol. 2004;122:1‐6. doi:10.1046/j.0022-202X.2003.22133.x 14962082

[58] Adler SG , Schwartz S , Williams ME , et al. Phase 1 study of anti‐CTGF monoclonal antibody in patients with diabetes and microalbuminuria. Clin J Am Soc Nephrol. 2010;5:1420‐1428. doi:10.2215/cjn.09321209 20522536 PMC2924405

[59] Johnson BG , Ren S , Karaca G , et al. Connective tissue growth factor domain 4 amplifies fibrotic kidney disease through activation of LDL receptor‐related protein 6. J Am Soc Nephrol. 2017;28:1769‐1782. doi:10.1681/asn.2016080826 28130402 PMC5461793

[60] Ng MR , Brugge JS . A stiff blow from the stroma: collagen crosslinking drives tumor progression. Cancer Cell. 2009;16:455‐457. doi:10.1016/j.ccr.2009.11.013 19962663

[61] Jiang Y , Zhang H , Wang J , et al. Targeting extracellular matrix stiffness and mechanotransducers to improve cancer therapy. J Hematol Oncol. 2022;15:34. doi:10.1186/s13045-022-01252-0 35331296 PMC8943941

[62] Ros M , Sala M , Saltel F . Linking matrix rigidity with EMT and cancer invasion. Dev Cell. 2020;54:293‐295. doi:10.1016/j.devcel.2020.06.032 32781020

[63] Saraswathibhatla A , Indana D , Chaudhuri O . Cell‐extracellular matrix mechanotransduction in 3D. Nat Rev Mol Cell Biol. 2023;24(7):495‐516. doi:10.1038/s41580-023-00583-1 36849594 PMC10656994

[64] Wei SC , Fattet L , Tsai JH , et al. Matrix stiffness drives epithelial‐mesenchymal transition and tumour metastasis through a TWIST1‐G3BP2 mechanotransduction pathway. Nature Cell Biology. 2015;17:678‐688. doi:10.1038/ncb3157 25893917 PMC4452027

[65] Calvo F , Ege N , Grande‐Garcia A , et al. Mechanotransduction and YAP‐dependent matrix remodelling is required for the generation and maintenance of cancer‐associated fibroblasts. Nat Cell Biol. 2013;15:637‐646. doi:10.1038/ncb2756 23708000 PMC3836234

[66] Rice AJ , Cortes E , Lachowski D , et al. Matrix stiffness induces epithelial‐mesenchymal transition and promotes chemoresistance in pancreatic cancer cells. Oncogenesis. 2017;6:e352. doi:10.1038/oncsis.2017.54 28671675 PMC5541706

[67] Richeldi L , Fernández Pérez ER , Costabel U , et al. Pamrevlumab, an anti‐connective tissue growth factor therapy, for idiopathic pulmonary fibrosis (PRAISE): a phase 2, randomised, double‐blind, placebo‐controlled trial. Lancet Respir Med. 2020;8:25‐33. doi:10.1016/S2213-2600(19)30262-0 31575509

[68] Ramazani Y , Knops N , Elmonem MA , et al. Connective tissue growth factor (CTGF) from basics to clinics. Matrix Biol. 2018;68–69:44‐66. doi:10.1016/j.matbio.2018.03.007 29574063

