# Supplementary material for: H. pylori‐induced NF‐κB‐PIEZO1‐YAP1‐CTGF axis drives gastric cancer progression and cancer‐associated fibroblast‐mediated tumour microenvironment remodelling
Source: Clin Transl Med. 2023 Nov 20;13(11):e1481. doi: 10.1002/ctm2.1481 (PMC10659770; doi:10.1002/ctm2.1481)
Supplement: Supplementary file 1 — Supporting Information [file CTM2-13-e1481-s001.docx]

Supplemental materials for

***H. pylori*-induced NF-κB-PIEZO1-YAP1-CTGF axis drives gastric cancer progression and cancer-associated fibroblast-mediated tumor microenvironment remodeling**

**
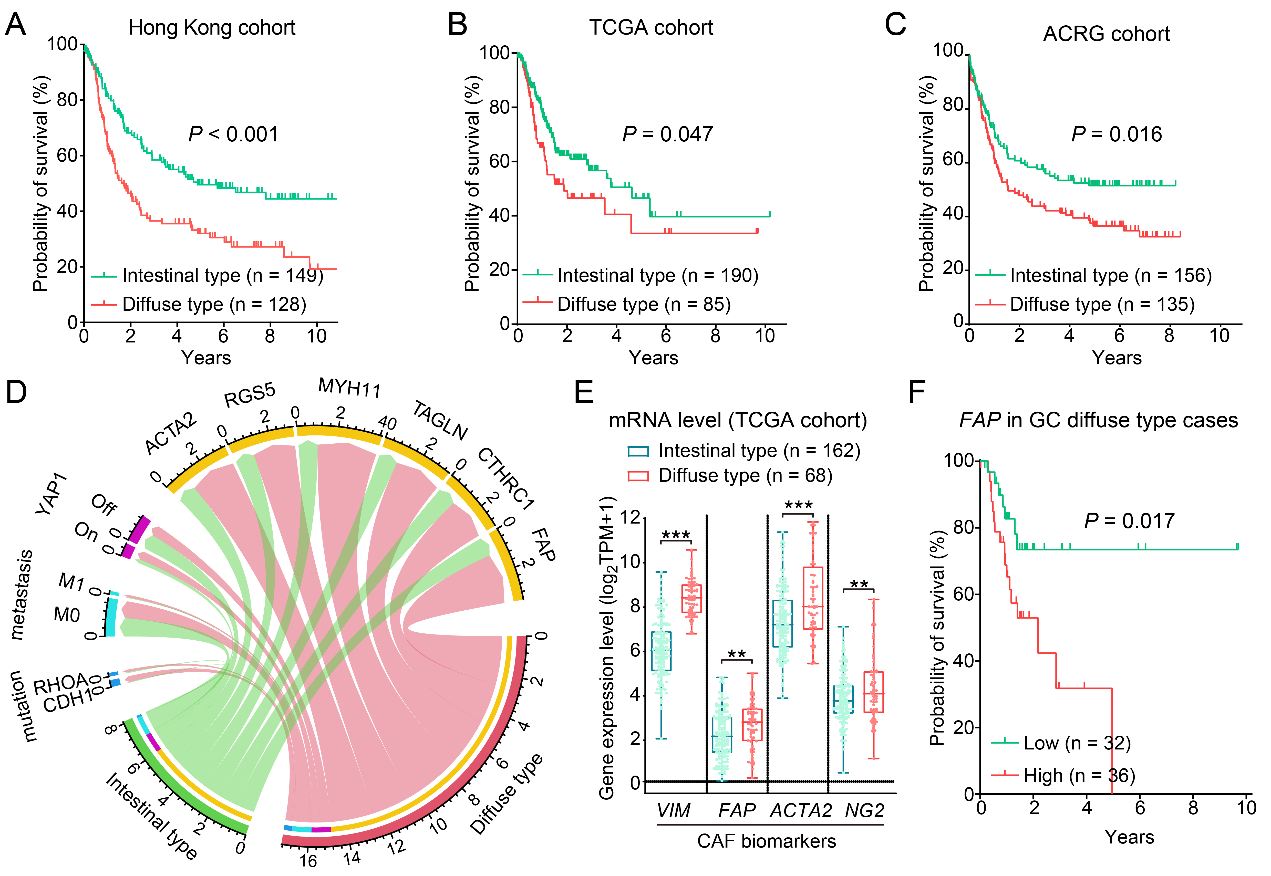
**

**Figure S1. Diffuse type GC was associated with unfavorable clinical outcomes and CAF signature.** (A-C) The diffuse type GC was associated with poor prognosis compared with the intestinal type GC in multiple cohorts (Hong Kong, TCGA, and ACRG cohorts). (D) The correlation of YAP1 pathway, CAF signature genes, metastasis, and *RHOA*/*CDH1* mutation status with intestinal or diffuse type GC. (E) The expression of CAF biomarkers *VIM*, *FAP*, *α-SMA*, and *NG2* in intestinal and diffuse types (**, *P* < 0.01; ***, *P* < 0.001). (F) The high expression of FAP was associated with poor prognosis in diffuse type GC cases (*P* = 0.017).

**
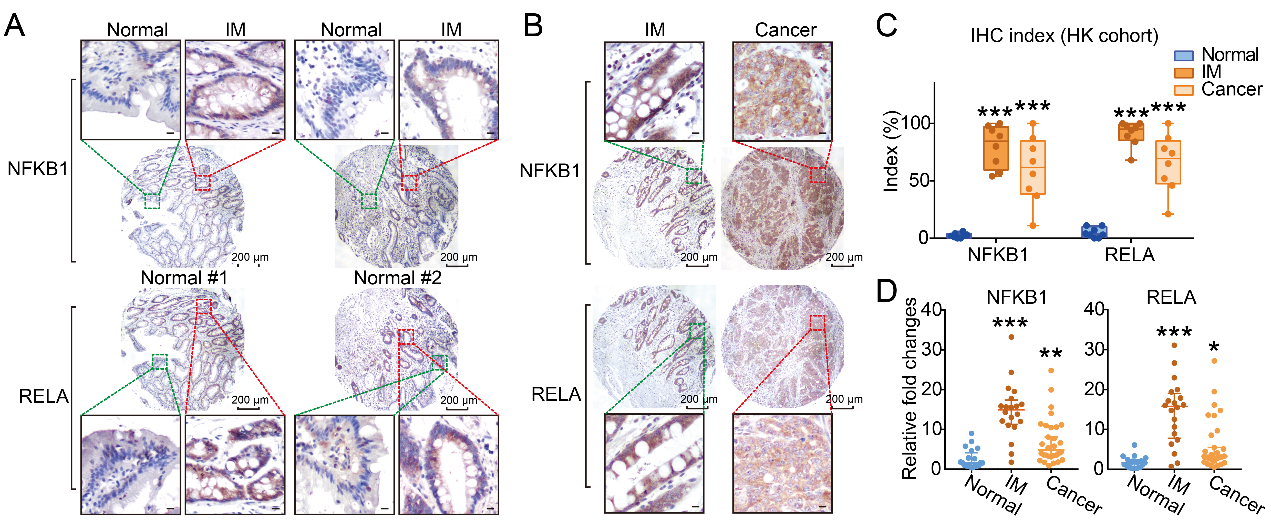
**

**Figure S2. NFKB1/RELA is highly expressed in IM and GC.** (A) The protein levels of NFKB1 and RELA in the normal gastric epithelial cells and paired IM. (B-C) NFKB1 and RELA were elevated in IM and GC from protein level by IHC scoring (***, *P* < 0.001). (D) The mRNA expression of *NFKB1* and *RELA* in the normal gastric epithelium, IM, and GC (*, *P* < 0.05; **, *P* < 0.01; ***, *P* < 0.001).


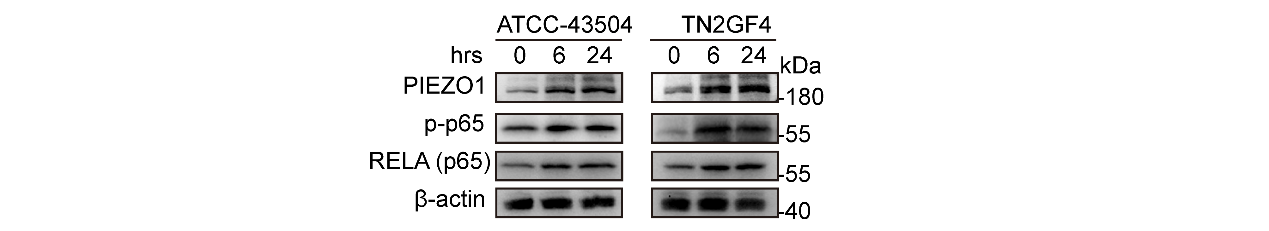


**Figure S3. PIEZO1, p-p65 expression in GSE1 after 24hrs treatment with *H. pylori* treatment.** Western Blot results indicated that 24-hour *H. pylori* treatment increased the phosphorylation levels of p65 and PIEZO1 expression in the GSE1 cell line.


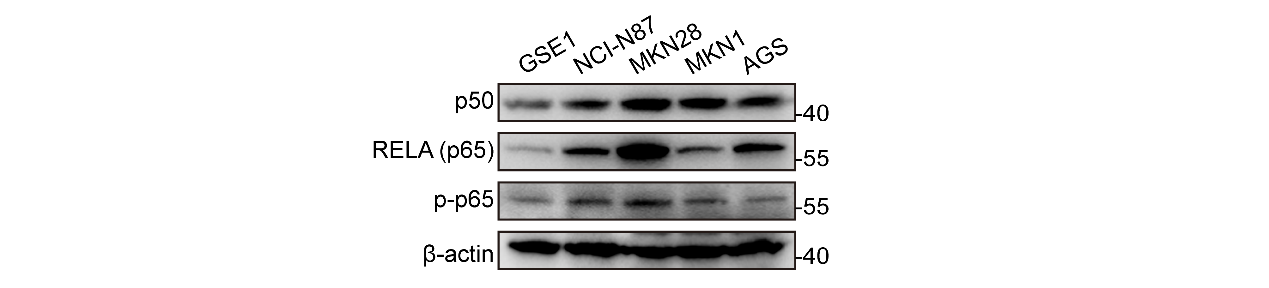


**Figure S4. Expression of p50, p65, p-p65 in normal gastric epithelial cell line and common gastric cancer cell lines.** WB analysis showed that the expression of p50, p65, p-p65 of GSE1 in normal gastric epithelial cell lines was significantly lower than that in gastric cancer cell lines compared with gastric cancer cells. Moreover, in gastric cancer cell lines, the expression of p50, p65, p-p65 was relatively higher in NCI-N87 and MKN28 cell lines.

**
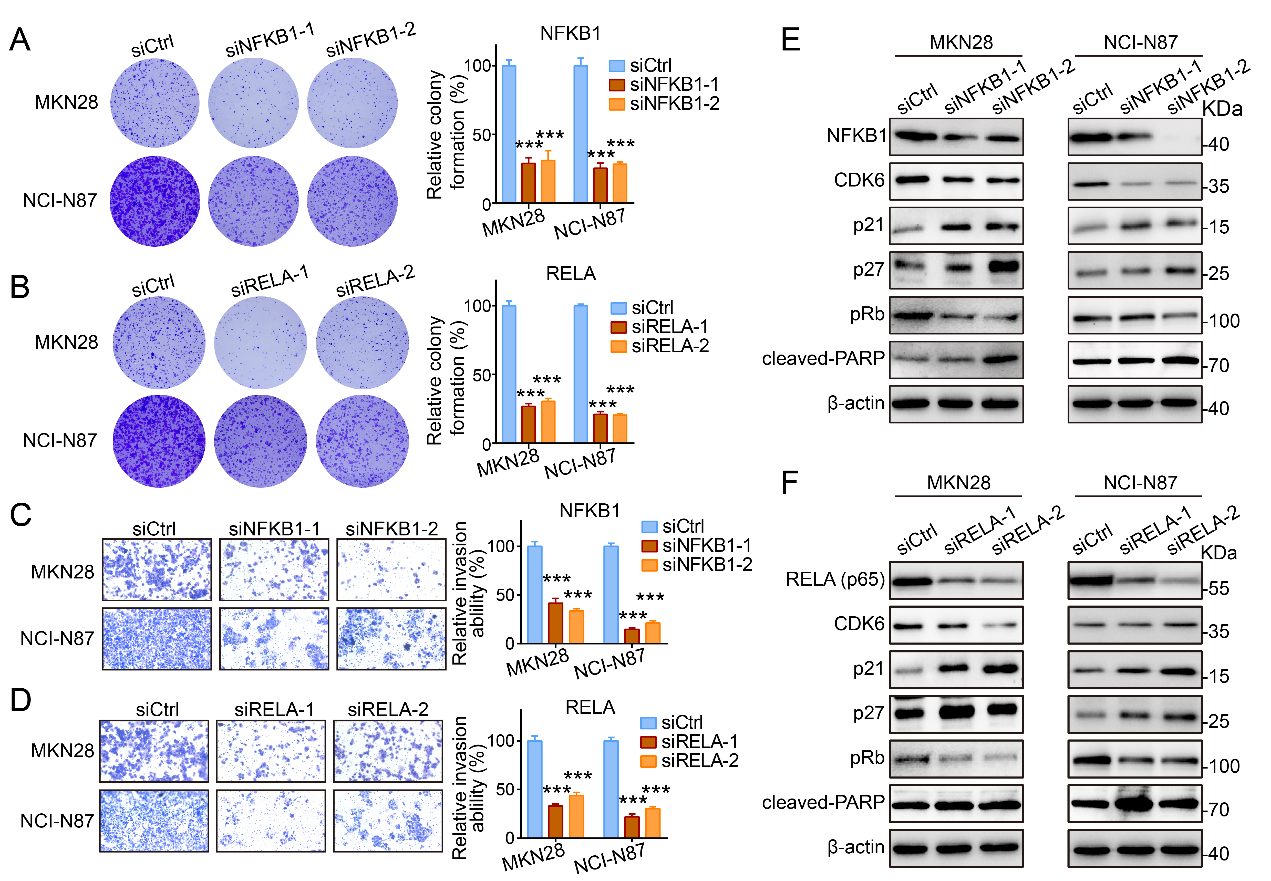
**

**Figure S5. NFKB1/RELA depletion exerts anti-tumor effects in GC.** (A-B) Knocking down NFKB1/RELA exerted an anti-tumor effect by suppressing monolayer colony formation. (C-D) NFKB1/RELA depletion impaired GC cell invasion. Outcomes are representative of three independent experiments. (E-F) Western blot analysis of cell-cycle and apoptosis-related biomarkers after NFKB1/RELA knockdown in MKN28 and NCI-N87 cells. Outcomes represent three independent experiments (***, *P* < 0.001).

**
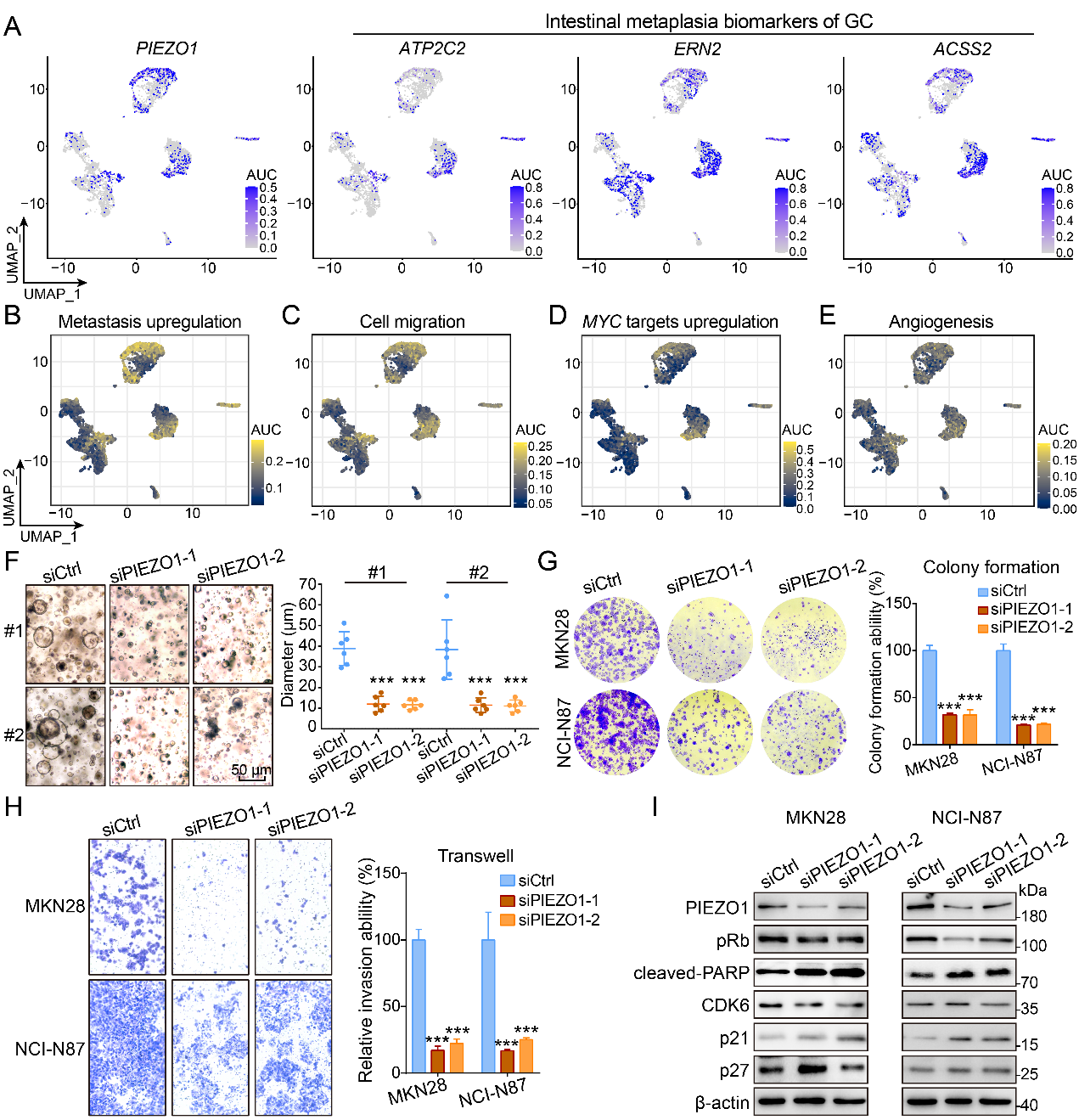
**

**Figure S6.** **PIEZO1 is highly expressed in IM and GC.** (A) The expression levels of *PIEZO1* and IM goblet cell markers *ATP2C2*, *ERN2*, and *ACSS2* were enriched in the same GC cell population. (B-E) The expression of *PIEZO1* was associated with metastasis upregulation, cell migration, Myc target upregulation, and angiogenesis. (F-H) Knocking down PIEZO1 suppressed patient-derived organoid growth, inhibited monolayer formation, and impaired GC cell invasion. (I) Western blot analysis of cell-cycle and apoptosis-related biomarkers after PIEZO1 knockdown in GC cells. Outcomes represent three independent experiments (***, *P* < 0.001).

**
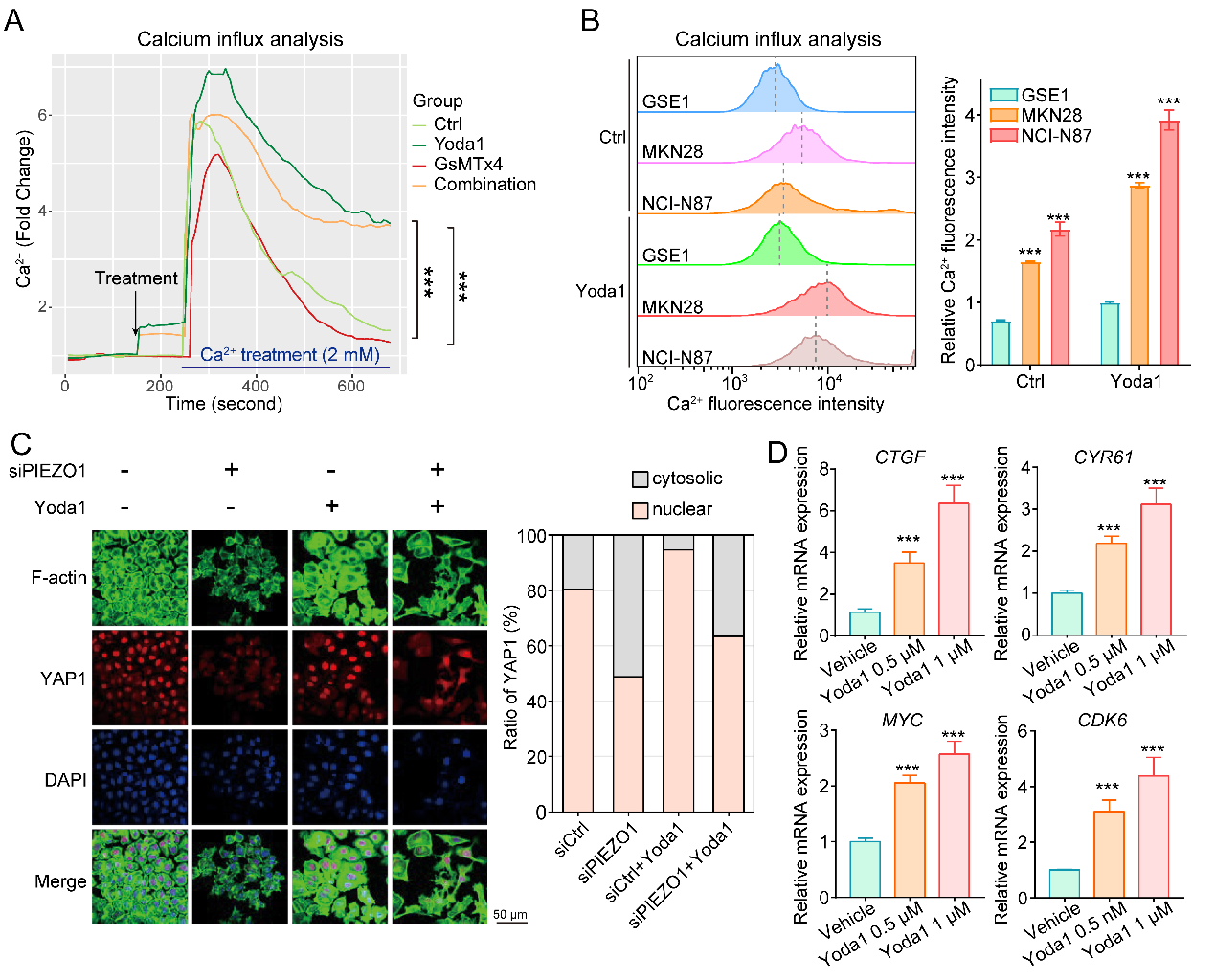
**

**Figure S7. PIEZO1 activates YAP1 signaling.** (A) Yoda1 (a PIEZO1 agonist) stimulation promoted the Ca^2+^ influx, but the stimulatory effect was partially abolished by the GsMTx4 (a PIEZO1 antagonist) treatment. (B) Flow analysis further revealed that stimulation with Yoda1 significantly enhanced the Ca^2+^ influx, with the GC cell lines MKN28 and N87 exhibiting a markedly higher degree of Ca^2+^ influx compared to the normal gastric epithelial cell line GSE1. (C)Yoda1 administration enhanced the YAP1 nuclear accumulation in NCI-N87 cells, while PIEZO1 depletion quenched the YAP1 nuclear translocation. (D) Yoda1 stimulation upregulated the classic YAP1 signature genes, *CTGF*, *CYR61*, *MYC,* and *CDK6*. Outcomes represent three independent experiments (***, *P* < 0.001).


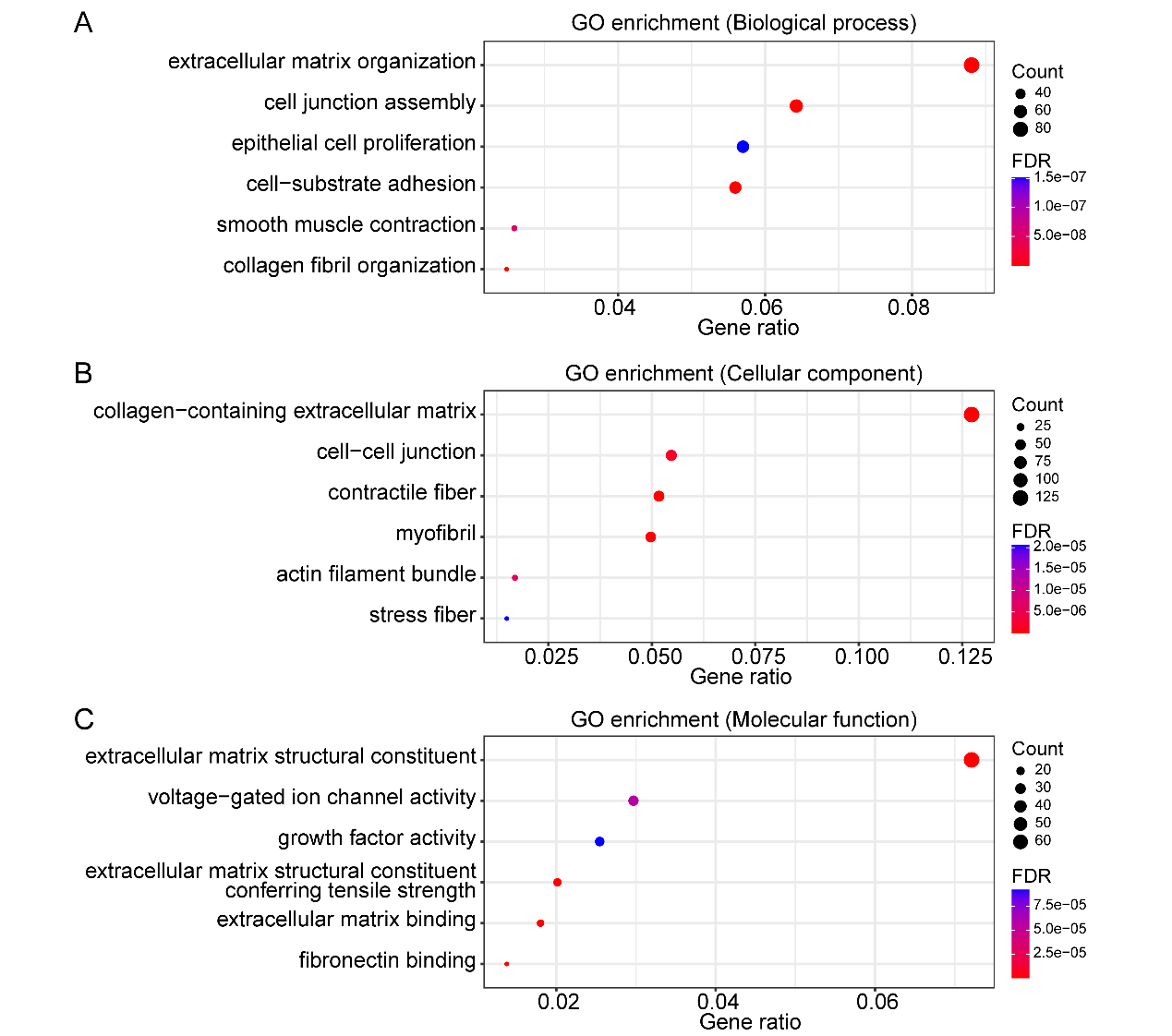


**Figure S8. GO enrichment analysis of differentially expressed genes in gastric cancer samples with low and high CTGF expression from the TCGA cohort.** (A) Pathways related to biological processes; (B) Pathways associated with cellular components; (C) Pathways connected to molecular functions.

**
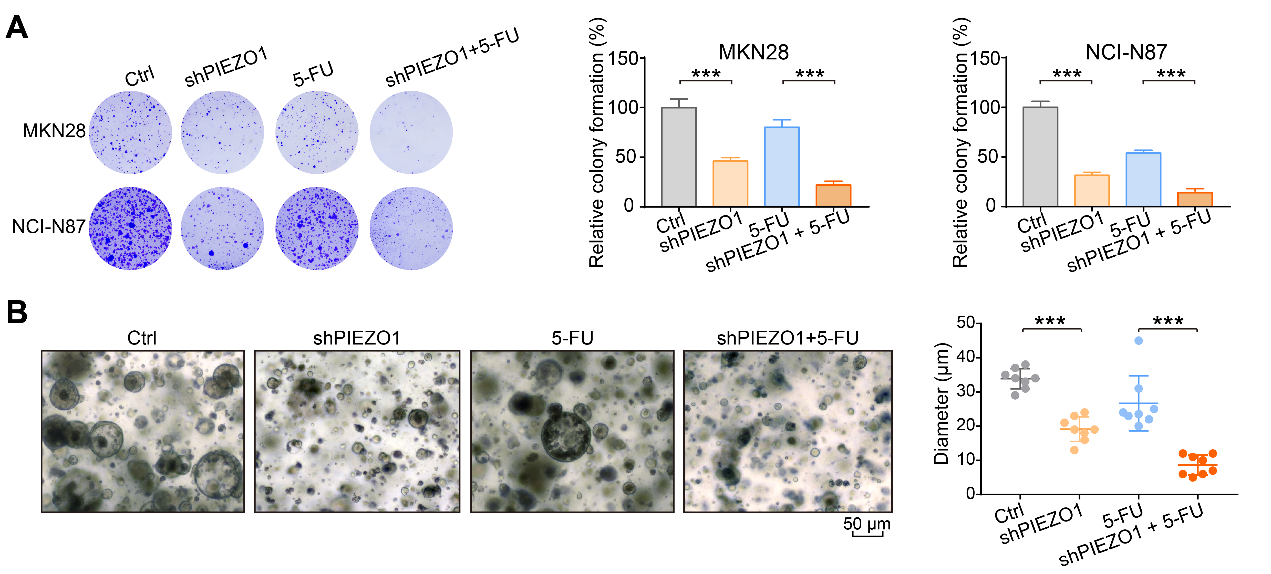
**

**Figure S9. Targeting PIEZO1 enhances the sensitivity of GC cells to 5-FU.** (A) PIEZO1 knockdown synergized with 5-FU to suppress cell growth in MKN28 and NCI-N87 cell lines (***, *P* < 0.001). (B) In a patient-derived organoid model, the synergistic effect of shPIEZO1 and 5-FU in suppressing tumor growth was also observed (***, *P* < 0.001).

**Table S1.** Dataset resources.

| **Dataset** | **Application** | **Resources** | **Website** |
| --- | --- | --- | --- |
| TCGA | RNA-Seq | Public data | http://cancergenome.nih.gov/ |
| ACRG (GSE62254) | mRNA expression | Public data | https://www.ncbi.nlm.nih.gov/geo/query/acc.cgi?acc=GSE62254 |
| GSE5081 | RNA-Seq | Public data | https://www.ncbi.nlm.nih.gov/geo/query/acc.cgi?acc=GSE5081 |
| scRNA-seq GC dataset | scRNA-seq | Public data | https://dna-discovery.stanford.edu/research/datasets/ |
| GSM2419824 | ChIP-seq | Public data | https://www.ncbi.nlm.nih.gov/geo/query/acc.cgi?acc=GSM2419824 |
| GSE228690 | RNA-Seq | In-house data | https://www.ncbi.nlm.nih.gov/g eo/query/acc.cgi?acc=GSE228690 |
| GSE228691 | RNA-Seq | In-house data | https://www.ncbi.nlm.nih .gov/g eo/query/acc.cgi?acc=GSE228691 |

**Table S2.** Antibodies for immunohistochemistry staining.

| **Antibody** | **Dilution** | **RRID** | **Company** |
| --- | --- | --- | --- |
| PIEZO1 | 1:500 | 15939-1-AP | Proteintech |
| α-SMA | 1:200 | ab5694 | Abcam |
| YAP1 | 1:50 | ab52771 | Abcam |
| Ki-67 | 1:200 | ab15580 | Abcam |
| NFKB1 (p105/p50) | 1:50 | #3035 | Cell Signaling Technology |
| RELA (p65) | 1:50 | #3034 | Cell Signaling Technology |
| cleaved-PARP (Asp214) | 1:50 | #9541 | Cell Signaling Technology |
| CTGF | 1:50 | #86641 | Cell Signaling Technology |
| DAPI | 1:10 | FP1490 | AKOYA BIOSCIENCES |
| HRP Rabbit Anti-Mouse IgG | 1:1000 | P0260 | Dako |
| HRP Goat Anti-Rabbit IgG | 1:2000 | P0448 | Dako |

**Table S3.** Antibodies for Western blot analysis.

| **Antibody** | **Dilution** | **RRID** | **Company** |
| --- | --- | --- | --- |
| pLATS1/2 (Ser909, Ser872) | 1:1000 | AB_2664907 | Thermo Fisher |
| pYAP1 (Ser127) | 1:1000 | #4911 | Cell Signaling Technology |
| CTGF | 1:1000 | #86641 | Cell Signaling Technology |
| CDK6 | 1:2000 | #3136 | Cell Signaling Technology |
| CDK4 | 1:1000 | #12790S | Cell Signaling Technology |
| p21 | 1:1000 | #2947 | Cell Signaling Technology |
| p27 | 1:1000 | #3688 | Cell Signaling Technology |
| RELA (p65) | 1:1000 | #3034 | Cell Signaling Technology |
| p-p65 | 1:1000 | sc-33039 | Santa Cruz Biotechnology |
| NF-κB1 (p105/p50) | 1:1000 | #3035 | Cell Signaling Technology |
| CCND1 | 1:1000 | #55506 | Cell Signaling Technology |
| CCND3 | 1:1000 | #2936 | Cell Signaling Technology |
| pRb (Ser807/811) | 1:1000 | #9308 | Cell Signaling Technology |
| cleaved-PARP (Asp214) | 1:1000 | #9541 | Cell Signaling Technology |
| GAPDH | 1:1000 | #2118 | Cell Signaling Technology |
| β-actin | 1:5000 | YM3028 | Immunoway |
| YAP1 | 1:5000 | ab52771 | Abcam |
| CYR61 | 1:1000 | #14479 | Cell Signaling Technology |
| c-Myc | 1:1000 | #9402 | Cell Signaling Technology |
| HRP Rabbit Anti-Mouse IgG | 1:2000 | P0260 | Dako |
| HRP Goat Anti-Rabbit IgG | 1:2000 | P0448 | Dako |

**Table S4.** Antibodies for immunofluorescence staining.

| **Antibody** | **Dilution** | **RRID** | **Company** |
| --- | --- | --- | --- |
| PIEZO1 | 1:100 | 15939-1-AP | Proteintech |
| YAP1 | 1:100 | ab52771 | Abcam |
| F-actin | 1:100 | ab112125 | Abcam |
| DAPI | 1:10 | FP1490 | AKOYA BIOSCIENCES |
| Alexa Fluor 555-Goat anti-Rabbit IgG (H+L) | 1:500 | AB_2535851 | Thermo Fisher |
| Alexa Fluor 488-Goat anti-Rabbit IgG (H+L) | 1:500 | AB_143165 | Thermo Fisher |

**Table S5.** Antibody for multiplex immunohistochemistry staining.

| **Antibody** | **Dilution** | **RRID** | **Company** |
| --- | --- | --- | --- |
| PIEZO1 | 1:100 | 15939-1-AP | Proteintech |
| α-SMA | 1:150 | ab5694 | Abcam |
| YAP1 | 1:100 | ab52771 | Abcam |
| DAPI | 1:10 | FP1490 | AKOYA BIOSCIENCES |
| Opal 520 Reagent | 1:100 | OP001001 | AKOYA BIOSCIENCES |
| Opal 620 Reagent | 1:100 | OP001004 | AKOYA BIOSCIENCES |
| Opal 690 Reagent | 1:100 | OP001006 | AKOYA BIOSCIENCES |
| Formerly Opal Polymer HRP Ms + Rb | 1:200 | ARH1001EA | AKOYA BIOSCIENCES |

**Table S6.** Primers used for qRT-PCR in this study.

| **Gene** | **Forward (5’-3’)** | **Forward (5’-3’)** |
| --- | --- | --- |
| NFKB1 | GGCAGCACTACTTCTTGACC | CAGCAAACATGGCAGGCTAT |
| RELA | GCCTGTCCTTTCTCATCCCA | CTGCCAGAGTTTCGGTTCAC |
| PIEZO1 | GGACTCTCGCTGGTCTACCT | GGGCACAATATGCAGGCAGA |
| CTGF | CAGCATGGACGTTCGTCTG | AACCACGGTTTGGTCCTTGG |
| CYR61 | GGTCAAAGTTACCGGGCAGT | GGAGGCATCGAATCCCAGC |
| CDK6 | AGTCTGATTACCTGCTCCGC | TCCAGAATCATTGCACCTGAG |
| MYC | GGCTCCTGGCAAAAGGTCA | CTGCGTAGTTGTGCTGATGT |
| B2M | ACTCTCTCTTTCTGGCCTGG | ATGTCGGATGGATGAAACCC |

**Table S7.** Antibodies uesd for ChIP-qPCR.

| **Antibody** | **Dilution** | **RRID** | **Company** |
| --- | --- | --- | --- |
| RELA (p65) | 1:50 | #3034 | Cell Signaling Technology |
| IgG | 1:50 | #2729 | Cell Signaling Technology |

**Table S8.** Primers used for PIEZO1 ChIP-qPCR in this study.

| **Gene** | **Forward (5’-3’)** | **Forward (5’-3’)** |
| --- | --- | --- |
| PIEZO1 | TCTCACTCTCACCGTCTCAC | GACTTTCGCTTTGCTCCCC |

**Table S9.** The oligonucleotide sequences were subcloned into the pGL3-basic plasmid for luciferase tests. Wild type, the full length of the transcription factor putative binding site; Mutation, the binding site was mutated; BS, binding site.

| **Transcription factor** | **Target** | **Type** | **Sequence (5’-3’)** |
| --- | --- | --- | --- |
| NF-κB | PIEZO1 | Wild type-BS | GCCGCGGGAACTCCGAGCCT |
| NF-κB | PIEZO1 | Mutation-BS | GCCGCAAAGGTCTTAAGCCT |

**Table S10.** Potential drug candidates targeting CTGF by computer-aided drug design (CADD, Ki < 10^-7^, n = 172).

| **Product name** | **CAS number** | **Molecular weight** | **Binding energy (kcal/mol)** | **Clinical information** |
| --- | --- | --- | --- | --- |
| Procyanidin C1 | 37064-30-5 | 866.77 | -14.05 | No Report |
| Theaflavin 3,3'-digallate | 30462-35-2 | 868.7 | -12.3 | No Report |
| Theaflavin-3'-gallate | 28543-07-9 | 716.6 | -12.25 | No Report |
| Theaflavin-3-gallate | 30462-34-1 | 716.6 | -11.94 | No Report |
| Tegatrabetan | 1227637-23-1 | 588.74 | -11.55 | No Report |
| Corilagin | 23094-69-1 | 634.45 | -11.45 | No Report |
| Momordin Ic | 96990-18-0 | 764.94 | -11.44 | No Report |
| Asiaticoside | 16830-15-2 | 959.12 | -11.32 | No Report |
| Amentoflavone | 1617-53-4 | 538.46 | -11.21 | No Report |
| Thonningianin A | 271579-11-4 | 874.71 | -11.05 | No Report |
| Lin28-let-7a antagonist 1 | 2024548-03-4 | 583.59 | -10.83 | No Report |
| Birabresib | 202590-98-5 | 491.99 | -10.83 | Phase 2 |
| Oroxin B | 114482-86-9 | 594.52 | -10.83 | No Report |
| Isoginkgetin | 548-19-6 | 566.51 | -10.82 | No Report |
| Saikosaponin A | 20736-09-8 | 780.98 | -10.8 | No Report |
| Sennoside B | 128-57-4 | 862.74 | -10.8 | No Report |
| Morusin | 62596-29-6 | 420.45 | -10.75 | No Report |
| Ginsenoside Rb1 | 41753-43-9 | 1109.29 | -10.73 | No Report |
| Procyanidin A2 | 41743-41-3 | 576.5 | -10.72 | No Report |
| AMG PERK 44 | 1883548-84-2 | 561.07 | -10.67 | No Report |
| Ginsenoside Re | 52286-59-6 | 947.15 | -10.67 | Phase 1 |
| Proanthocyanidins | 20347-71-1 | 594.52 | -10.65 | Phase 4 |
| CDDO-Im | 443104-02-7 | 541.72 | -10.61 | No Report |
| 5-IAF | 63368-54-7 | 515.25 | -10.61 | No Report |
| Hypericin | 548-04-9 | 504.44 | -10.6 | Phase 1 |
| Epothilone D | 189453-10-9 | 491.68 | -10.59 | Phase 2 |
| Ginkgetin | 481-46-9 | 566.51 | -10.58 | No Report |
| Hederacolchiside A1 | 106577-39-3 | 897.1 | -10.52 | No Report |
| Pseudoginsenoside RT1 | 98474-74-9 | 927.08 | -10.51 | No Report |
| CX-5461 | 1138549-36-6 | 513.61 | -10.49 | Phase 1 |
| Lonafarnib | 193275-84-2 | 638.82 | -10.44 | Launched |
| Withaferin A | 5119-48-2 | 470.6 | -10.39 | No Report |
| Bardoxolone | 218600-44-3 | 491.66 | -10.38 | Phase 3 |
| Procyanidin B2 | 29106-49-8 | 578.52 | -10.38 | No Report |
| NHWD-870 | 2115742-03-3 | 491.59 | -10.37 | No Report |
| Mogroside IV-A | 88901-41-1 | 1125.29 | -10.33 | No Report |
| Oligomycin A | 579-13-5 | 791.06 | -10.32 | No Report |
| Quinestrol | 152-43-2 | 364.52 | -10.3 | Launched |
| Puerarin | 3681-99-0 | 416.38 | -10.3 | Launched |
| EC359 | 2012591-09-0 | 540.68 | -10.29 | No Report |
| Orientin | 28608-75-5 | 448.38 | -10.29 | No Report |
| VU0661013 | 2131184-57-9 | 712.66 | -10.28 | No Report |
| CDDO-2P-Im | 1883650-96-1 | 618.81 | -10.25 | No Report |
| PMX-53 | 219639-75-5 | 896.09 | -10.24 | No Report |
| SHIN1 | 2146095-85-2 | 400.47 | -10.24 | No Report |
| Zotarolimus | 221877-54-9 | 966.21 | -10.23 | Phase 4 |
| CDDO-3P-Im | 1883650-95-0 | 618.81 | -10.23 | No Report |
| GS-626510 | 1637770-13-8 | 394.47 | -10.22 | No Report |
| CDDO-EA | 932730-51-3 | 518.73 | -10.21 | No Report |
| BET-BAY 002 | 1588521-78-1 | 403.86 | -10.21 | No Report |
| Isoschaftoside | 52012-29-0 | 564.49 | -10.21 | No Report |
| MD2-TLR4-IN-1 | 2249801-12-3 | 421.28 | -10.2 | No Report |
| MRTX-1257 | 2206736-04-9 | 565.71 | -10.18 | No Report |
| Pictilisib | 957054-30-7 | 513.64 | -10.17 | Phase 2 |
| Bardoxolone methyl | 218600-53-4 | 505.69 | -10.16 | Phase 3 |
| PIK-294 | 900185-02-6 | 489.53 | -10.16 | No Report |
| Ganoderic acid A | 81907-62-2 | 516.67 | -10.15 | No Report |
| S55746 | 1448584-12-0 | 710.82 | -10.13 | Phase 1 |
| CIL56 | 300802-28-2 | 489.61 | -10.13 | No Report |
| Borussertib | 1800070-77-2 | 596.68 | -10.12 | No Report |
| Punicalagin | 65995-63-3 | 1084.72 | -10.12 | Phase 4 |
| ALK2-IN-2 | 2254409-25-9 | 497.61 | -10.1 | No Report |
| Maytansinol | 57103-68-1 | 565.06 | -10.09 | No Report |
| Epothilone B | 152044-54-7 | 507.68 | -10.07 | Phase 3 |
| VR23 | 1624602-30-7 | 477.88 | -10.06 | No Report |
| Rottlerin | 1982/8/6 | 516.54 | -10.06 | No Report |
| 20(R)-Ginsenoside Rg3 | 38243-03-7 | 785.01 | -10.05 | No Report |
| Tauroursodeoxycholate | 14605-22-2 | 499.7 | -10.04 | No Report |
| FGFR1/DDR2 inhibitor 1 | 2308497-58-5 | 501.5 | -10.03 | No Report |
| CD437 | 125316-60-1 | 398.49 | -10.02 | No Report |
| ELN-441958 | 913064-47-8 | 501.02 | -10.02 | No Report |
| Eriocitrin | 13463-28-0 | 596.53 | -10 | No Report |
| Complanatuside | 116183-66-5 | 624.54 | -10 | No Report |
| 10-Deacetyltaxol | 78432-77-6 | 811.87 | -9.99 | No Report |
| IPR-803 | 892243-35-5 | 453.49 | -9.99 | No Report |
| NPS-1034 | 1221713-92-3 | 551.54 | -9.98 | No Report |
| BMX-IN-1 | 1431525-23-3 | 524.59 | -9.97 | No Report |
| (+)-JQ-1 | 1268524-70-4 | 456.99 | -9.96 | No Report |
| Estradiol (cypionate) | 313-06-4 | 396.56 | -9.96 | Launched |
| LY2090314 | 603288-22-8 | 512.53 | -9.95 | Phase 2 |
| AZ9482 | 1825345-33-2 | 450.49 | -9.93 | No Report |
| Polyphyllin VI | 55916-51-3 | 738.9 | -9.93 | No Report |
| (-)-Epigallocatechin Gallate | 989-51-5 | 458.37 | -9.92 | Phase 4 |
| AMI-1 (free acid) | 134-47-4 | 504.49 | -9.92 | No Report |
| γ-Oryzanol | 11042-64-1 | 602.89 | -9.92 | Launched |
| Raddeanin A | 89412-79-3 | 897.1 | -9.92 | No Report |
| TBAP-001 | 1777832-90-2 | 531.51 | -9.91 | No Report |
| Plicamycin | 18378-89-7 | 1085.15 | -9.91 | Launched |
| Brassinolide | 72962-43-7 | 480.68 | -9.91 | No Report |
| CU-CPT17e | 2109805-75-4 | 504.49 | -9.91 | No Report |
| Rutin | 153-18-4 | 610.52 | -9.9 | Launched |
| Ginsenoside F1 | 53963-43-2 | 638.87 | -9.9 | No Report |
| A-1331852 | 1430844-80-6 | 658.81 | -9.89 | No Report |
| SCH772984 | 942183-80-4 | 587.67 | -9.89 | No Report |
| Demethylzeylasteral | 107316-88-1 | 480.59 | -9.89 | No Report |
| ONO-7475 | 1646839-59-9 | 562.57 | -9.88 | Phase 1 |
| Adavivint | 1467093-03-3 | 505.55 | -9.88 | Phase 3 |
| 42-(2-Tetrazolyl)rapamycin | 221877-56-1 | 966.21 | -9.87 | No Report |
| Temoporfin | 122341-38-2 | 680.75 | -9.87 | Launched |
| Ziyuglycoside I | 35286-58-9 | 766.96 | -9.87 | No Report |
| IACS-010759 | 1570496-34-2 | 562.56 | -9.87 | No Report |
| MX69 | 1005264-47-0 | 474.57 | -9.86 | No Report |
| Gossypol | 303-45-7 | 518.55 | -9.85 | Launched |
| Deltarasin | 1440898-61-2 | 603.75 | -9.85 | No Report |
| Icariin | 489-32-7 | 676.66 | -9.85 | Phase 3 |
| VER-246608 | 1684386-71-7 | 552.96 | -9.84 | No Report |
| Inauhzin | 309271-94-1 | 469.58 | -9.84 | No Report |
| ABBV-744 | 2138861-99-9 | 491.55 | -9.83 | Phase 1 |
| (-)-Gallocatechin gallate | 4233-96-9 | 458.37 | -9.83 | No Report |
| MAT2A inhibitor 1 | 2201057-10-3 | 526.61 | -9.82 | No Report |
| Omaveloxolone | 1474034-05-3 | 554.71 | -9.81 | Phase 2 |
| Rheb inhibitor NR1 | 2216763-38-9 | 578.3 | -9.81 | No Report |
| 10074-G5 | 413611-93-5 | 332.31 | -9.8 | No Report |
| IQ 1 | 331001-62-8 | 362.42 | -9.8 | No Report |
| WEHI-9625 |  | 593.71 | -9.79 | No Report |
| CEP-40783 | 1437321-24-8 | 588.56 | -9.78 | Phase 1 |
| 4E1RCat | 328998-25-0 | 478.45 | -9.77 | No Report |
| BMS-986158 | 1800340-40-2 | 495.62 | -9.76 | Phase 2 |
| Liquiritin | 551-15-5 | 418.39 | -9.76 | No Report |
| Digitonin | 11024-24-1 | 1229.31 | -9.76 | No Report |
| Epibrassinolide | 78821-43-9 | 480.68 | -9.75 | No Report |
| Gambogenic acid | 173932-75-7 | 630.77 | -9.75 | No Report |
| Oleandrin | 465-16-7 | 576.72 | -9.72 | No Report |
| Elacridar (hydrochloride) | 143851-98-3 | 600.1 | -9.72 | No Report |
| Geraniin | 60976-49-0 | 952.64 | -9.72 | No Report |
| RBC8 | 361185-42-4 | 424.45 | -9.71 | No Report |
| Trifolirhizin | 6807-83-6 | 446.4 | -9.71 | No Report |
| Scutellarin | 27740-01-8 | 462.36 | -9.71 | No Report |
| Pennogenin 3-O-beta-chacotrioside | 55916-52-4 | 885.04 | -9.71 | No Report |
| TAS0728 | 2088323-16-2 | 504.58 | -9.71 | Phase 2 |
| SU11274 | 658084-23-2 | 568.09 | -9.7 | No Report |
| Phillyrin | 487-41-2 | 534.55 | -9.7 | Launched |
| Centrinone | 1798871-30-3 | 633.65 | -9.69 | No Report |
| KIRA6 | 1589527-65-0 | 518.53 | -9.69 | No Report |
| Apigenin-7-glucuronide | 29741-09-1 | 446.36 | -9.69 | No Report |
| CX-6258 | 1202916-90-2 | 461.94 | -9.68 | No Report |
| Geldanamycin | 30562-34-6 | 560.64 | -9.67 | No Report |
| Astragaloside IV | 84687-43-4 | 784.97 | -9.67 | No Report |
| Neriifolin | 466-07-9 | 534.68 | -9.67 | No Report |
| TVB-3166 | 1533438-83-3 | 384.47 | -9.66 | No Report |
| Paris saponin VII | 68124-04-9 | 1031.18 | -9.66 | No Report |
| Isosilybin | 72581-71-6 | 482.44 | -9.65 | No Report |
| A-196 | 1982372-88-2 | 359.25 | -9.64 | No Report |
| Cot inhibitor-2 | 915363-56-3 | 539.43 | -9.64 | No Report |
| Proscillaridin A | 466-06-8 | 530.65 | -9.64 | No Report |
| dBET1 | 1799711-21-9 | 785.27 | -9.63 | No Report |
| BET bromodomain inhibitor | 1505453-59-7 | 445.9 | -9.63 | No Report |
| Hesperidin | 520-26-3 | 610.56 | -9.62 | Launched |
| GW788388 | 452342-67-5 | 425.48 | -9.62 | No Report |
| MS645 | 2250091-96-2 | 938.04 | -9.61 | No Report |
| WYE-132 | 1144068-46-1 | 519.6 | -9.61 | No Report |
| Torin 1 | 1222998-36-8 | 607.62 | -9.6 | No Report |
| YM-201636 | 371942-69-7 | 467.48 | -9.6 | No Report |
| FASN-IN-3 | 2097262-60-5 | 384.47 | -9.6 | No Report |
| Galunisertib | 700874-72-2 | 369.42 | -9.59 | Phase 3 |
| MY-5445 | 78351-75-4 | 331.8 | -9.59 | No Report |
| Candesartan | 139481-59-7 | 440.45 | -9.59 | Launched |
| Aloin | 1415-73-2 | 418.39 | -9.59 | No Report |
| Timosaponin AIII | 41059-79-4 | 740.92 | -9.59 | No Report |
| SB-633825 | 956613-01-7 | 483.58 | -9.59 | No Report |
| Epothilone A | 152044-53-6 | 493.66 | -9.58 | No Report |
| Kaempferitrin | 482-38-2 | 578.52 | -9.58 | No Report |
| SM 16 | 614749-78-9 | 430.5 | -9.58 | No Report |
| Metarrestin | 1443414-10-5 | 474.6 | -9.57 | Phase 1 |
| Ursodiol | 128-13-2 | 392.57 | -9.57 | Launched |
| ML228 | 1357171-62-0 | 415.49 | -9.56 | No Report |
| Galeterone | 851983-85-2 | 388.55 | -9.56 | Phase 3 |
| Dioscin | 19057-60-4 | 869.04 | -9.56 | Launched |
| Rebastinib | 1020172-07-9 | 553.59 | -9.55 | Phase 2 |
| NVP-BHG712 | 940310-85-0 | 503.48 | -9.55 | No Report |
| Asiaticoside B | 125265-68-1 | 975.12 | -9.55 | No Report |
| Enzastaurin | 170364-57-5 | 515.61 | -9.55 | Phase 3 |

**Table S11.** The clinicopathological characteristics of GC samples in Figure 1A.

| **Characteristic** | **Case #2** | **Case #5** | **Case #53** | **Case #59** | **Case #64** | **Case #78** | **Case #79** | **Case #99** |
| --- | --- | --- | --- | --- | --- | --- | --- | --- |
| **Gender/Age (yr)** | Female (74) | Male (69) | Male (76) | Female (43) | Male (76) | Female (54) | Female (69) | Male (55) |
| **Grade** | 2 | 2 | 3 | 3 | 3 | 2 | 3 | 2 |
| **Clinical stage** | Ⅲ | Ⅱ | Ⅲb | Ⅳ | Ⅳ | Ⅲa | Ⅳ | Ⅰ |
| **LN** | 1 | 0 | 1 | 1 | 1 | 1 | 1 | 0 |
| **TNM stage** | T3N2M0 | T3N0M0 | T3N2M0 | T3N2M1 | T3N3M0 | T3N1M0 | T3N3M0 | T2N0M0 |
| ***H. pylori* infection** | Positive | Negative | Negative | Positive | Negative | Positive | Positive | Negative |
| **Lauren classification** | Intestinal | Intestinal | Diffuse | Intestinal | Diffuse | Intestinal | Diffuse | Intestinal |
| **Overall survival (month)** | 2.6 | 30.7 | 8.4 | 6.0 | 12.0 | 29.8 | 11.9 | 58.6 |

**Table S12.** GO enriched pathways of the Yoda1-treatment group compared with the control group in the NCI-N87 cells.

| **Ontology** | **ID** | **Description** | **Gene ratio** | **Count** | ***P* value** | ***Q* value** |
| --- | --- | --- | --- | --- | --- | --- |
| BP | GO:0030198 | extracellular matrix organization | 0.038 | 55 | 1.55E-05 | 0.020141299 |
| BP | GO:0048730 | epidermis morphogenesis | 0.008 | 11 | 1.63E-05 | 0.020141299 |
| BP | GO:0043062 | extracellular structure organization | 0.038 | 55 | 1.66E-05 | 0.020141299 |
| BP | GO:0035082 | axoneme assembly | 0.012 | 17 | 1.79E-05 | 0.020141299 |
| BP | GO:0050910 | detection of mechanical stimulus involved in sensory perception of sound | 0.005 | 7 | 1.86E-05 | 0.020141299 |
| BP | GO:0001578 | microtubule bundle formation | 0.014 | 21 | 2.55E-05 | 0.02295641 |
| CC | GO:0098862 | cluster of actin-based cell projections | 0.019 | 29 | 1.99E-05 | 0.011481153 |
| CC | GO:0034703 | cation channel complex | 0.022 | 34 | 0.000149565 | 0.028635882 |
| CC | GO:0031225 | anchored component of the membrane | 0.018 | 28 | 0.000193385 | 0.028635882 |
| CC | GO:0034702 | ion channel complex | 0.027 | 42 | 0.000215914 | 0.028635882 |
| CC | GO:1902495 | transmembrane transporter complex | 0.028 | 44 | 0.00028768 | 0.028635882 |
| CC | GO:0016324 | apical plasma membrane | 0.031 | 48 | 0.000297855 | 0.028635882 |
| CC | GO:0062023 | collagen-containing extracellular matrix | 0.035 | 54 | 0.000455103 | 0.032775328 |
| CC | GO:1990351 | transporter complex | 0.028 | 44 | 0.000490631 | 0.032775328 |
| CC | GO:0031526 | brush border membrane | 0.008 | 13 | 0.000511367 | 0.032775328 |
| CC | GO:0045177 | apical part of cell | 0.035 | 54 | 0.00063592 | 0.033429082 |
| CC | GO:0005903 | brush border | 0.012 | 19 | 0.000637471 | 0.033429082 |
| CC | GO:0031091 | platelet alpha granule | 0.011 | 17 | 0.000742289 | 0.035681953 |
| MF | GO:0005261 | cation channel activity | 0.036 | 53 | 1.70E-06 | 0.000998033 |
| MF | GO:0005244 | voltage-gated ion channel activity | 0.024 | 36 | 3.12E-06 | 0.000998033 |
| MF | GO:0022832 | voltage-gated channel activity | 0.024 | 36 | 3.12E-06 | 0.000998033 |
| MF | GO:0005216 | ion channel activity | 0.042 | 62 | 6.57E-06 | 0.00157841 |
| MF | GO:0048018 | receptor ligand activity | 0.045 | 67 | 1.28E-05 | 0.002022287 |
| MF | GO:0015267 | channel activity | 0.044 | 66 | 1.41E-05 | 0.002022287 |
| MF | GO:0022803 | passive transmembrane transporter activity | 0.044 | 66 | 1.51E-05 | 0.002022287 |
| MF | GO:0030546 | signaling receptor activator activity | 0.045 | 67 | 1.79E-05 | 0.002022287 |
| MF | GO:0022843 | voltage-gated cation channel activity | 0.018 | 27 | 1.89E-05 | 0.002022287 |
| MF | GO:0022836 | gated channel activity | 0.034 | 50 | 2.19E-05 | 0.002108527 |
| MF | GO:0005262 | calcium channel activity | 0.015 | 22 | 0.000181572 | 0.01586366 |
| MF | GO:0005540 | hyaluronic acid binding | 0.005 | 8 | 0.000207552 | 0.016622353 |
| MF | GO:0046873 | metal ion transmembrane transporter activity | 0.038 | 56 | 0.000245054 | 0.017376647 |
| MF | GO:0008083 | growth factor activity | 0.018 | 27 | 0.000253132 | 0.017376647 |
| MF | GO:0038024 | cargo receptor activity | 0.010 | 15 | 0.000648905 | 0.041575486 |
| MF | GO:0048019 | receptor antagonist activity | 0.006 | 9 | 0.000742781 | 0.044615747 |

**Table S13.** The clinicopathological characteristics of the GC samples in Figure 5A.

| **Characteristic** | **Case #183** | **Case #194** | **Case #198** |
| --- | --- | --- | --- |
| **Gender/Age (yr)** | Male (55) | Male (65) | Female (65) |
| **Grade** | 2 | 3 | 3 |
| **Clinical stage** | Ⅳ | Ⅰb | Ⅲb |
| **LN** | 1 | 0 | 1 |
| **TNM stage** | T2N2M1 | T2N0M0 | T3N2M0 |
| ***H. pylori* infection** | Positive | Positive | Positive |
| **Lauren classification** | Intestinal | Intestinal | Intestinal |
| **Overall survival (month)** | 52.4 | 79.1 | 41.7 |
